# Metabolite Neu5Ac triggers SLC3A2 degradation promoting vascular endothelial ferroptosis and aggravates atherosclerosis progression in ApoE^-/-^mice

**DOI:** 10.7150/thno.87968

**Published:** 2023-09-04

**Authors:** Peng Xiang, Qingqiu Chen, Le Chen, Jin Lei, Zhiyi Yuan, Hui Hu, Yining Lu, Xianmin Wang, Tingting Wang, Ruihong Yu, Wanping Zhang, Jun Zhang, Chao Yu, Limei Ma

**Affiliations:** 1 College of Pharmacy, Chongqing Medical University, 400010, Chongqing, China.; 2 Chongqing Key Laboratory for Pharmaceutical Metabolism Research, 400010, Chongqing, China.; 3 Xi'an No.1 Hospital, The First Affiliated Hospital of Northwest University, Xi'an, 710002, Shaanxi, China.

**Keywords:** atherosclerosis, endothelial inflammatory injury, Neu5Ac, ferroptosis, SLC3A2

## Abstract

**Background:** Atherosclerosis (AS) is still the major cause of cardiovascular disease (CVD) as well as stroke. Endothelial metabolic disorder has been found to be activated and then promote endothelial cells (ECs) injury, which is regarded to initiate AS progression. N-acetylneuraminic acid (Neu5Ac), a metabolite produced by hexosamine-sialic acid pathway branching from glucose metabolism, was presented as a notable biomarker of CVD and is positively correlated with ECs function. However, few studies explain whether Neu5Ac regulate AS progression by affecting EC function as well as its involved mechanisms are still unknown.

**Methods:** Here, we mimicked an animal model in *ApoE^-/-^* mice which displaying similar plasma Neu5Ac levels with AS model to investigate its effect on AS progression.

**Results:** We found that Neu5Ac exacerbated plaques area and increased lipids in plasma in absence of HFD feeding, and ECs inflammatory injury was supposed as the triggering factor upon Neu5Ac treatment with increasing expression of IL-1β, ICAM-1, and promoting ability of monocyte adhesion to ECs. Mechanistic studies showed that Neu5Ac facilitated SLC3A2 binding to ubiquitin and then triggered P62 mediated degradation, further leading to accumulation of lipid peroxidation in ECs. Fer-1 could inhibit ECs injury and reverse AS progression induced by Neu5Ac in *ApoE^-/-^* mice. Interestingly, mitochondrial dysfunction was also partly participated in ECs injury after Neu5Ac treatment and been reversed by Fer-1.

**Conclusions:** Together, our study unveils a new mechanism by which evaluated metabolite Neu5Ac could promote SLC3A2 associated endothelial ferroptosis to activate ECs injury and AS plaque progression, thus providing a new insight into the role of Neu5Ac-ferroptosis pathway in AS. Also, our research revealed that pharmacological inhibition of ferroptosis may provide a novel therapeutic strategy for premature AS.

## Introduction

Atherosclerosis (AS) is a chronic inflammatory vascular disease, the leading cause of cardiovascular disease (CVD) 1-3, and endothelial cells (ECs) injury is crucial in AS progression and displayed as essential step to initiate vascular injury 4. Recent studies have insinuated altered ECs metabolism disorder as new features of endothelial dysfunction 5, 6, which will release risk metabolites such as lactate, α-ketoglutarate, fumarate, 2-hydroxyglutarate and others to regulate cell physiology and affect vascular homeostasis 7, 8. Therefore, investigating the intrinsic biological activity of such metabolites may provide new therapeutic targets for vascular injury disease.

N-acetylneuraminic acid (Neu5Ac), a kind of negatively charged nine-carbon monosaccharide, is a distinct metabolite from glucose- hexosamine metabolism disorder at the level of fructose-6-phosphate 9. Several rate-limiting enzymes may be responsible for Neu5Ac metabolism homeostasis, such as glutamine fructose-6-phosphate transaminase 1 (GFAT1) catalyzes glucose branch to HBP pathway, then UDP-N-acetylglucosamine 2-epimerase/N-acetylmannosamine-kinase (GNE) as well as N-acetylneuraminic acid synthase (NANS) starts sialic acids synthesis in cytosol 10, 11. And CMP-sialic acid synthetase (CMAS) activates Neu5Ac to CMP-Neu5Ac, which is prerequisite for the sialylation of cell surface glycoconjugates 12. Further neuraminidase (NEUs) cleave terminal sialic acid residues from glycoproteins and plays an important role in Neu5Ac dynamic equilibrium 13. Numerous studies have found that Neu5Ac was observed highly accumulated in patient's plasma during vascular injury disease, such as cancer 14, diabetes 15, and atherosclerosis 16. Such enzymes like NEUs are strongly up-regulated and involved in pathogenesis of AS 17. However, no evidence showed that metabolite Neu5Ac itself will regulate vascular function and then contribute to AS progression.

Vascular endothelium liminal forms a single cell layer separating blood from the vessels. Its surface is reported to be heavily covered with Neu5Ac, which is essential to protect against inflammatory cells adhesion to ECs and then maintain vascular healthy homeostasis 18, 19. Strategies of inhibiting Neu5Ac release from ECs displayed well organized protection on ECs function as well as AS progression 16, 20. In our present study, we also noticed that Neu5Ac level was increased upon ECs inflammatory stimulus. While inhibition of Neu5Ac synthesis or cleavage by using GFAT1 inhibitor or NEUs inhibitor could attenuate EC dysfunction, which further suggesting that ECs injury was positively correlated with Neu5Ac metabolism disorder. However, the further involved regulation mechanism still comprehensively unknows.

Ferroptosis is a typical form of iron-dependent cell death driven by the accumulation of lipid peroxides. And glutathione peroxidase 4 (GPX4), the unique member of the glutathione peroxidase family, shields cell membranes from peroxidative damage under stress stimulation 21. Numerous studies have shown that ferroptosis is a potential target for anti-AS strategy 22. GPX4 inactivation could cause accumulation of phospholipid hydroperoxides, which then contributes to the essential process of plaque formation, including vascular endothelial dysfunction and foam cell formation 23. Therefore, inhibition of GPX4 could be identified as a notable maker of ferroptosis activation in AS. Recently, studies showed Neu5Ac molecules participated in the dysfunctional cation metabolism and eventual ferroptosis in the SARS-CoV-2 infected cells 24, which suggested that Neu5Ac may be involved in the occurrence of ferroptosis pathway. Further clinical studies found that decreasing of plasma GPX4 levels is accompanied with increasing of Neu5Ac levels in acute coronary syndrome patients compared to normal subjects 25, which further indicated that Neu5Ac strongly correlated with GPX4 and may participated in ferroptosis process. However, the devoted mechanism of ferroptosis associated with Neu5Ac in ECs injury is still unknown.

In the present study, we used the *ApoE^-/-^* mice, a well-recognized lipoprotein metabolism disorder and inflammatory mice model 26, to detect the potential effect of elevated Neu5Ac on lipids accumulation and inflammation both *in vivo* and *in vitro*. We found that Neu5Ac aggravated plaques formation and induced the lipid disorder in the absence of HFD feeding, which indicated that elevated Neu5Ac in vascular displayed as a risk factor of AS. Mechanistically, we first found that Neu5Ac directly triggered endothelial ferroptosis and then induced EC inflammation. SLC3A2, an important mediator of ferroptosis, was facilitated by Neu5Ac to ubiquitination degradation, which further contributed to ferroptosis pathway. In sum, these data identified Neu5Ac as a risk factor to vascular homeostasis which induced ECs ferroptosis and then strongly aggravated ECs inflammatory injury. Our present work also represented a promising therapeutic strategy for the treatment of ECs injury disease, such as AS.

## Materials and methods

### Reagents and antibodies

The following antibodies were used: anti-GAPDH (100242-MM05, Sino Biologica, China), anti-β-Actin (sc-47778, Santa Cruz Biotechnology, USA), anti-SLC7A11 (A2413, ABclonal, China, China), ati-SLC3A2 (A19880, ABclonal, China), anti-GPX4 (A11243, ABclonal, China), anti-GPX4 (A1933, ABclonal, China), anti-GPX4 (67763-1-Ig, Proteintech, USA), anti-TFRC (A5865, ABclonal, China), anti-ICAM-1 (A19300, ABclonal, China), anti-VCAM-1 (A0279, ABclonal, China), anti-IL-1β (A19636, ABclonal, China), anti-Parkin (A0968, ABclonal, China), anti-TOM20 (A19403, ABclonal, China). Neu5Ac (purity: >95%, #HY-10400) was purchased from MCE (China), apoptosis inhibitor Z-VAD-FMK (C1202-0.02ml, Beyotim, China), ferroptosis inhibitor Ferrostatin-1 (T6500, Topscience, USA), ferroptosis inducer Erastin (E1765, Topscience, USA) and P-3Fax-Neu5Ac (#5760/10) were obtained from R&D Systems (USA).

### Human serum sample

Serum of hyperlipidemia and healthy donors were obtained from University-Town Hospital of Chongqing Medical University. Patients with hypertension or diabetes and other metabolic diseases are excluded. All described human experiments were in accordance with the national guidelines and approved by the Ethics Committee of University-town Hospital of Chongqing Medical University (approval number: NO. LL-202256).

### Animal models

Eight-week-old male *ApoE^-/-^* mice (*C57BL/6J* background, 20~25 g) were purchased from Beijing Vital River Laboratory Animal Technology Co., Ltd (license number: SCXK(Jing)2021-0006). Ten-week-old male *C57BL/6J* mice (25~30 g) were obtained from the Experimental Animal Center of Chongqing Medical University (approval numbers: IACUC-CQMU-2022-0002). This study was approved by the Ethics Committee of the Animal Ethical and Experimental Committee of Chongqing Medical University. Mice were housed in specific pathogen-free rooms with temperature (20-26 ℃) and humidity (50-60%). During the experiment, mice had free access to food and water. After a week of adaptive feeding, the *C57BL/6J* mice were randomly divided into 2 groups, the model group (CFD, n = 5, normal saline treatment group), the Neu5Ac group (CFD, n = 5, 60 mg/kg/d by intraperitoneal injection), and the *ApoE^-/-^* mice were randomly divided into 5 groups, two model groups (CFD, HFD, n = 8, normal saline treatment group), Neu5Ac group (CFD, n = 5, 60 mg/kg/d by intraperitoneal injection), Neu5Ac&Fer-1 group (CFD, n = 6, 60 mg/kg/d N-eu5Ac and 1 mg/kg/d Fer-1 by intraperitoneal injection) and HFD&Fer-1(HFD, n = 5, 1 mg/kg/d Fer-1 by intraperitoneal injection). Fer-1 concentration and mode of administration refer to reported studies 27. At the end of the study, mice were anesthetized and sacrificed. The vasculature was perfused completely with sterile phosphate-buffered saline (PBS)-heparin sodium solution by cardiac puncture to wash out blood from the heart and all vessels. Hearts were excised and fixed in 4% paraformaldehyde. Aorta, liver, and kidney were collected in a sterile PBS solution for further use.

### Atherosclerotic lesion analysis

Aortas were obtained from mice, then fixed in 4% paraformaldehyde solution and stained with Oil-red O (Solarbio, China) to quantify the lesion area. Paraffin-embedded aortas were cut into 6-8 μm thick slides for Hematoxylin-Eosin (HE)staining and Masson staining to visualize the necrotic core areas and the collagen contents. Images of plaques were captured under a Leica microscope, and quantitative analysis was involved using Image-Pro Plus (Media Cybernetics, USA). For immunostaining, aorta sections were fixed in ice-cold acetone for 10 min, washed with PBS blocked with normal goat serum for 1 h at room temperature, then incubated with primary antibody at 4 ℃ overnight. Sections were rinsed with PBS, incubated with secondary antibodies for 1 h at room temperature, then mounted with flourished mounting medium with DAPI (C1005, Beyotime, China,). Images were captured under a Leica confocal laser scanning microscopy.

### Detection of lipid levels

Collected blood samples were loaded into Eppendorf tubes containing sodium heparin. After centrifugation, plasma was separated. Plasma total cholesterol (CHO), triglyceride (TG), high-density lipoprotein cholesterol (HDL-C), and low-density lipoprotein cholesterol (LDL-C) levels were detected using a biochemical analyzer (Shenzhen Raytor Biotech Co. Ltd., China).

### Neu5Ac level analysis *in vivo* and *in vitro*

Neu5Ac in plasma was measured by Sialic Acid (SA) Colorimetric Assay Kit (E-BC-K068-M, Elabscience, China) according to the manufacturer's instructions. Extracted 25 μL of standard samples (Reagent 1) at different concentrations from standard tubes and 25 μL of serum/plasma from assay tubes into 2 mL EP tubes, and then add 500 μL of Reagent 2 to each of these tubes. Vortex mixing, 100 °C water bath for 15 min, cooling with running water, 2325 g centrifugation 10 min, supernatant 200 μL was added to the enzyme plate, and 560 nm was used to determine the OD value of each hole. The Neu5Ac levels in aortic sinus were detected by using WGA staining. Detailly, the fresh aortic sinus sections were incubated with WGA-FITC (1:200, GeneTEX, USA) for 1 h at room temperature. After DAPI staining, the images were captured under a confocal immunofluorescence microscopy. For Neu5Ac levels in cells, we collect the HUVECs medium after different treatment. Detailly, mix 100 μL of the sample with 300 μL of acetonitrile (1:3) by vortexing, centrifuge at 15,000 g for 10 min was used, and dry the supernatant at room temperature under vacuum for 1.5 h, then add 50 μL of ammonium acetate solution to re-dissolve, centrifuge for 10 min, add 40 μL of supernatant to 40 μL of ammonium acetate and mix well, remove 40 μL of the mixture for LC-MS testing.

### Cell culture

Human umbilical vein endothelial cells (HUVECs) were obtained from ScienCell Research Laboratories, Inc. (USA) and cultured in ECM medium supplemented with 5% fetal bovine serum, 1% growth factors, and 1% antibodies (penicillin/streptomycin) in a 37 °C, 5% CO_2_ incubator. HEK293 cells were gifted from Dr. Bo Chen and cultured in DMEM medium supplemented with 10% fetal bovine serum in a 37 °C, 5% CO_2_ incubator.

### Cell viability assay

For cell viability assays, HUVEC cells were seeded in 96-well plates at 1 × 10^5^/mL using Cell Count Kit 8 (CCK-8) (C0037, Beyotime, China) and incubated overnight at 37 °C. Then the cells were subjected to Neu5Ac treatment (0-40 mM) for 12 and 24 h, followed by the addition of CCK-8 reagent to the cell culture medium. After incubation for 3 h, absorbance values were measured at 450 nm and cell viability was calculated.

### Western blotting

Total proteins from the cultured cells were extracted using RIPA Lysis Buffer (P0013B, Beyotime, China,) containing PMSF (ST506, Beyotime, China,) and protease inhibitors. BCA assay kit (E-BC-K318-M, Elabscience) was used to determine the protein concentration. Proteins were subjected to SDS-PAGE and transferred onto PVDF (Millipore, USA). Following, antigen blocking with 5% BSA, the blots were incubated with primary antibodies overnight at 4 ℃, followed by secondary antibodies or HRP-labelled secondary antibodies at room temperature for 1 h. The final detection of immunoreactive bands was developed using an enhanced chemiluminescent Western blot system (ECL) (BL520A, biosharp China) with exposure to a chemiluminescence imaging system (UVP). The immunoblotting signal intensity was measured using ImageJ 64 software.

### Real-time quantitative PCR analysis

Total RNA from cells was extracted by the TRIzol method. The RNA was reverse transcribed by Evo M-MLV Mix Kit with gDNA Clean for qPCR (A4A0765, Accurate Biotechnol). Real-time PCR was performed with SYBR^®^ Green Realtime Master qPCR Mix (A4A0496, Accurate Biotechnol). The levels of ACTIN mRNA were used as endogenous control. The relative mRNA expression values were calculated by 2^-∆∆Ct^ methods.

The primers are listed as follows:

GAPDH: 5'-TGGTATCGTGGAAGGACTC-3' (Forward), 5'-AGTAGAGGCAGGGATGAGT-3' (Reverse), SLC7A11: 5'-TGCTGGGCTGATTTTATCTTCG-3' (Forward), 5'-GAAAGGGCAACCATGAAGAGG-3' (Reverse), SLC3A2: 5'-CTGGTGCCGTGGTCATAATC-3' (Forward), 5'-GCTCAGGTAATCGAGACGCC-3' (Reverse), ICAM-1: 5'-TCTTCCTCGGCCTTCCCATA-3' (Forward), 5'-AGGTACCATGGCCCCAAATG-3' (Reverse), IL-1β: 5'-TGGCAGAAAGGGAACAGAAA-3' (Forward), 5'-CTGGCTGATGGACAGGAGAT-3' (Reverse).

### Monocyte adhesion assay

The cells were cultured after a series of treatments, THP-1 cells were labeled with Calcein AM (C2012-0.1ml, Beyotime, China) and incubated on top of a monolayer of HUVECs for 1 h. Using phosphate-buffered saline washed Nonadherent cells 3 times. Monocyte adhesion was photographed using a Nikon inverted fluorescence microscopy.

### Immunofluorescence staining

HUVECs were seeded on the coverslips in 24-well plates and cultured overnight at 37 °C. Further, the cells were fixed with ice methanol for 15 min and then permeabilized with 0.1% Triton X-100 (T8200, Solarbio, China) and blocked with 5% BSA for 1 h. Then, cells were incubated with primary antibodies diluted in blocking buffer overnight at 4 °C. After PBS washed 3 times, the cells were incubated with secondary antibodies diluted in blocking solution for 1 h and DAPI for 10 min at room temperature. Cells were then washed 3 times with PBS, and the coverslips were sealed with nail polish. Images were captured by using a Leica confocal laser scanning microscopy. Fluorescence images were analyzed using ImageJ software.

Frozen sections of the aortic sinus were restored to room temperature and blocked with 10% BSA for 1 h. Then aortic sinus was incubated with primary antibodies diluted in PBS overnight at 4 ℃. After PBS washed 3 times, the aortic sinus was incubated with secondary antibodies diluted in PBS for 1 h and DAPI for 10 min at room temperature. Images were captured by using a Leica confocal laser scanning microscopy. The primary antibodies used in this study targeted anti-GPX4 (A1933, ABclonal, China), anti-GPX4 (67763-1-Ig, Proteintech), and anti-TFRC (A5865, ABclonal, China). The secondary antibodies were: FITC-labeled Goat Anti-Rabbit IgG (H+L) (A0562, Beyotim, China), Alexa Fluor 555-labeled Donkey Anti-mouse IgG (H+L) (A0460, Beyotime, China).

### Transcriptome sequencing analysis

Using TRIzol® Reagen extracted total RNA from the HUVECs according to the manufacturer's instructions (Invitrogen) and genomic DNA was removed using DNase I (Takara). Degradation and contamination of RNA was monitored on a 1% agarose gel. RNA quality was then determined using a 2100 Bioanalyzer (Agilent Technologies) and quantified using an ND-2000 (Nascent Technologies). Only high-quality RNA sample (OD 260/280 = 1.8~2.2, OD 260/230 ≥ 2.0, RIN ≥ 8.0, 28S:18S ≥ 1.0, > 1μg) was used to construct sequencing library.

For identifying DEGs (differentially expressed genes) between the two different groups, gene expression levels were calculated for each gene according to the transcripts per million reads (TPM) method. RSEM (http://deweylab.biostat.wisc.edu/rsem) was used to quantify gene abundance. Essentially, differential expression analysis was performed using the DESeq2/DEGseq/edgeR/Limma/NOIseq, DEGs with |log2 (fold change) | ≥ 1 and P-adjust ≤ 0.05 (DESeq2/edgeR/Limma) / P-adjust ≤ 0.001 (DEGseq) / Prob > 0.8 (NOIseq) were considered to be significantly different expressed genes. Additionally, analyses of functional enrichment including GO (Gene Ontology, http://www.geneontology.org) and KEGG (Kyoto Encyclopedia of the Genome, http://www.genome.jp/kegg/) were conducted to determine which DEGs were significantly enriched for GO terms and metabolic pathways when compared to the whole transcriptome background at P-adjust ≤ 0.05 were significantly enriched for GO terms and metabolic pathways. GO functional enrichment and KEGG pathway analysis were carried out by Goatools (https://github.com/tanghaibao/Goatools) and KOBAS (http://kobas.cbi.pku.edu.cn/).

### Ferroptosis markers assays in vitro

Cellular malondialdehyde (MDA) and glutathione (GSH) content were measured with Malondialdehyde (MDA) Test Kit (S0131S, Beyotime, China), Glutathione (GSH) Assay Kit (S0052, Beyotime, China) according to the manufacturer's instructions. Lipid peroxidation was analysis by using C11 BODIPY 581/591 (D3861, Thermo Fisher Scientific, USA) as a molecular probe. Briefly, the HUVECs were treated with Neu5Ac or Neu5Ac&Fer-1 for 24 h. The original medium was replaced by 1 mL medium containing 5 μM C11 BODIPY 581/591 dye, and the cells were cultured for an additional 30 min. At the end time point, cells were washed with PBS 3 times, then added with 2 mL medium without FBS, and finally observed under Leica confocal fluorescence microscope. Also, intracellular Fe^2+^ levels were detected by using Ferro Orange probe. Briefly, the Mito-Tracker Green fluorescent probe (C1048, Beyotime, China) was first added to the HUVECs for 30 min, then the previous solution was replaced by fresh FBSS. Subsequently, the prewarming Ferro Orange working solution (F374, Dojindo, Japan) was used to treat the cells kept in a Lucite place for 30 min. In addition, for detecting the level of ROS in vivo, fresh aortic sinus section was incubated with PBS containing 10 μM DCFH-DA at 37 ℃ for 30 min. Then washed with PBS 3 times and fluorescence intensity was determined with a confocal immunofluorescence microscopy at 488/525 nm.

### Transmission electron microscopy (TEM) assays

For the TEM assay, after trypsinization, HUVEC was centrifuged at 1200 rpm/min for 5 min and then fixed with 4% glutaraldehyde at 4 °C for 2 hours. Then, HUVEC were post-fixed with 1% osmium tetroxide at 4 °C for 1 hour. Subsequently, HUVEC were dehydrated with a range of alcohols and acetone and embedded in Epon 816. Sections were ultrathin and stained with uranyl acetate and lead citrate using a Leica ultramicrotome. TEM images were taken under a JEM-1400Puls transmission electron microscope.

### Evaluation of mitochondrial function

The Mitochondrial Membrane Potential Assay Kit with TMRE (C2001S, Beyotime, China) was used to detect mitochondrial dysfunction after Neu5Ac treatment. HUVECs was treated with above solution for 45 min, then the solution was replaced by fresh PBS. Subsequently, the prewarming Hoechst 33242 (C1026, Beyotime, China) working solution was used to treat the cells kept in a Lucite place for 1 h. In addition, mitochondrial superoxide level was also detected by using Mito-SOX assay kit. HUVECs were seeded in a 24-well lucifugal plate, then incubated with 5 mM Mito-SOX™ reagent solution for 10 min. The cells were washed two times by PBS, and the level of mitochondria ROS was assessed by Leica confocal fluorescence microscope, Ex/Em = 510/580 nm.

### Co-immunoprecipitation analysis

Whole-cell lysates were prepared from native lysis buffer containing a complete protease inhibitor cocktail. The supernatant was collected after centrifugation at 14,000 g for 15 min and incubated with specific antibodies for 12 h at 4 °C with constant rotation, followed by the addition of 50 μL of Protein A+G agarose beads (P2055, BIOTEK, China) and incubation with constant rotation at 4 °C for 4 h. The lysate was washed three times for 5 min each time, and then washed with the lysis buffer for 5 min each time. The beads were washed with lysis buffer 3 times for 5 min each. The precipitated proteins were then eluted from the beads by adding SDS-PAGE Sampling Buffer and boiling at 100 °C for 10 min. Immunoprecipitate or cell lysate samples are used for WB analysis.

### Geo dataset

We obtained CMAH expression values from the GEO database (http://www.ncbi.nlm.nih.gov/geo/). We used key words “Atherosclerosis”, “*ApoE^-/-^* mice”, and “*C57BL/6J mice*”. Eight gene expression datasets (GSE10000, GSE83112, GSE2372, GSE28125, GSE39264, GSE143533, GSE70126, GSE191044) were selected and downloaded to screen CMAH expression value.

### Atomic modeling analysis

The AlphaFold2 program was used to predict the 3D structures of SLC3A2 and P62, respectively. The highest scoring conformations were used for subsequent molecular docking, dynamic simulation, or analysis.

For molecular docking, we obtained the structure of Neu5Ac from the PDB database (PDB ID 4NFD). Subsequently, Neu5Ac was docked with the just predicted SLC3A2 and P62 using the ZDOCK online server (https://zdock.umassmed.edu/) to predict the binding model.

For molecular dynamics simulations, we applied the GROMACS software package (version 2021.03) to run conventional MD simulations to investigate the changes in the individual conformations of SLC3A2, Neu5Ac, and P62, as well as the binding modes of the SLC3A2-Neu5Ac, SLC3A2-P62, and SLC3A2-Neu5Ac-P62 complexes. We parameterized SLC3A2, P62, and Neu5Ac using the force fields amber14sb and OL15 ff, respectively. The TIP3P was used for the waters. Dissolve the protein or protein-Neu5Ac complex in an octahedral water box and add 0.150 M of chloride and sodium ions to neutralize the charge of the system. First, minimum system energy was minimized through 50,000 steps using the steepest descent minimization method. Second, restrictions were placed on the positions of the heavy atoms in order to run both a constant number of atoms, volume, and temperature (NVT) equilibrium and a constant number of atoms, pressure, and temperature (NPT) equilibrium within 50,000 steps. The system temperature was maintained at 300 K, and the system pressure was maintained at 105 pa. The system was considered well balanced at the desired temperature and pressure upon completion of these two equilibrium phases. Unconstrained simulations were performed for 100 ns. Tremendous energy and coordinate system of the trajectory were saved every 10 ps. ChimeraX and PyMOL were used to animate interactive patterns and dynamic trajectories in the simulated trajectories. To examine the conformational changes of SLC3A2, PCA was performed on molecular dynamics trajectories using Desmond to characterize the magnitude of the free energies of various conformations of the macromolecule.

### Free energy calculations

In our work, using gmx_MMPBSA (the MM-PB (GB) SA calculation tool from GROMACS) to calculate MM-GBSA, which has been widely used for the estimation of binding free energies in pharmaceutical research.

### Statistical analysis

Statistical comparisons between two independent groups were performed with Student's t-test while one-way ANOVA tests were used to evaluate statistical differences between the two conditions. The GraphPad Prism 8.0.2 software was used to analyze the data present as Means ± S.D. * *p* < 0.05; ** *p* < 0.01; *** *p* < 0.001.

## Results

### Metabolite Neu5Ac elevated in CVD patients' serum and accelerated atherosclerotic plaque progression in *ApoE^-/-^* mice

**Figure 1:**
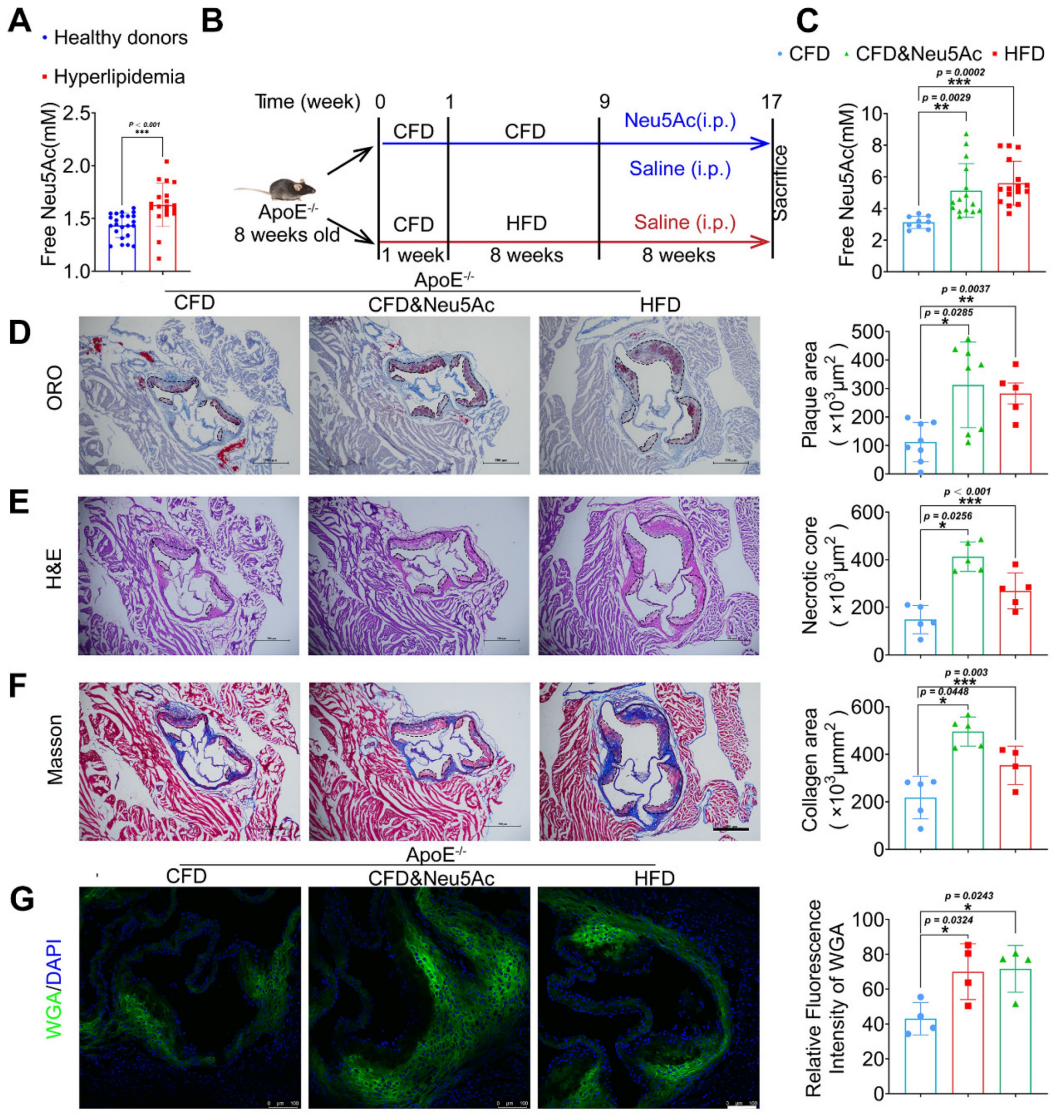
** Elevated Neu5Ac accelerates atherosclerotic plaque progression in ApoE^-/-^ mice.** (A) Plasma Neu5Ac levels were tested by using Assay Kit and found upregulated in Hyperlipidemic compared with healthy donors (n > 20 per group). (B) Experimental design for investigating the effect of Neu5Ac on AS progression in *ApoE^-/-^* mice. Briefly, *ApoE^-/-^* mice were fed with CFD or HFD and treated intraperitoneally with Neu5Ac (60 mg/kg/d) or saline (60 mg/kg/d) for 8 weeks. (C) Plasma Neu5Ac levels were found to be higher in mice with HFD or Neu5Ac-injecting than mice in CFD-fed group (n > 5 per group). (D) Representative microphotograph of Oil Red O-stained aortic sinus and showed that Oil Red O positive plaque area was larger in HFD-fed and Neu5Ac-injected groups than that in CFD-fed group. Scale bars = 500 μm (n > 3 per group). (E) Representative microphotograph of H&E-stained aortic sinus and found that necrotic core area was up-regulated in HFD or Neu5Ac treatment mice compared with CFD-fed mice. Scale bars = 500 μm (n > 3 per group). (F) Representative images of the aortic sinus stained with Masson's trichrome stain. The quantification of collagen area was larger in HFD or Neu5Ac treatment mice compared with mice in CFD-fed group. Scale bars = 500 μm (n > 3 per group). (G) Representative images of immunofluorescence staining of Neu5Ac-specific fluorescent dye WGA (green) in aortic sinus and found decreased immunofluorescence intensity of WGA in CFD mice compared with HFD and Neu5Ac mice, with quantitative data in the right panel. Scale bars = 100 μm (n = 4 per group). Statistical analysis was performed by Student *t* test for A, and one-way ANOVA followed by the Tukey test for C-G. Neu5Ac: N-acetylneuraminic acid, WGA: Wheat germ agglutinin, ORO: Oil Red O.

Clinical data show that metabolite Neu5Ac in circulation is a notable marker of CVD progression 28, 29, which is also confirmed in our present study that Neu5Ac level in hyperlipidemic was significantly increased compared with health donors (Figure 1A). To better understand the correlation of evaluated Neu5Ac with AS progress, we fed *ApoE^-/-^* mice with HFD to construct AS model (Figure 1B) and examined Neu5Ac levels in plasma as well as AS lesions. Our data showed that the level of Neu5Ac was increasing in *ApoE^-/-^* mice upon HFD fed for 8 weeks (Figure 1C), combined with the increased plaque size (Figure 1D), aggravated necrotic core (Figure 1E), as well as accumulated collagens in aortic sinus (Figure 1F). We also found that TC and LDL values were markedly increased in HFD-fed *ApoE^-/-^* mice (Figure S1B) compared with mice in CFD-fed group. In addition, HFD-fed induced obvious liver injury as evidenced by increased serum level of AST and ALT in mice (Figure S1C), but there were no significant changes in the kidney injury indicators (Figure S1D). These data obviously suggested that AS mice model was established successfully, and free Neu5Ac levels in serum were also increased along with the AS progression. Interestingly, we also noticed that the expression of Neu5Ac in plaque was increased upon AS model established by using WGA staining (Figure 1G). Together, these data indicated that metabolite Neu5Ac may be strongly associated with AS progression.

Next, in order to investigate the effect of Neu5Ac itself on AS progression, we established a model by using Neu5Ac intraperitoneal injection for 8 weeks, which was detected with similar plasma and plaque concentration of Neu5Ac as AS model we established before (Figure 1B-C and Figure 1G). Then we tested the similar index which indicating AS progression. As shown in Figure 1D-F, we found that Neu5Ac increased atherosclerotic plaque size, necrotic core area as well as collagen fibrosis area even in the absence of HFD feeding in *ApoE^-/-^*mice. Biochemical analysis of serum lipids also demonstrated that Neu5Ac increased the TC, TG, LDL levels but showed no overt difference on HDL level as well as body weight compared with CFD control group (Figure S1A-B). In addition, results from liver function indicator (AST, ALT) and histological analysis (Figure S1C and Figure S1E) showed that Neu5Ac increased the levels of ALT, AST and induced unregular morphology compared with those in control group, indicating that Neu5Ac may induce liver injury. Meanwhile, we observed no obvious variations on the kidney function indicator (UREA, CREA, UA). However, kidney histological analysis manifested that Neu5Ac caused vacuolar-like degradation appearance (Figure S1D and Figure S1F).

Further, in order to verify the unusual predictive effect of Neu5Ac on AS progression, we also generated a model in *C57BL/6J* mice with intraperitoneal injection of Neu5Ac in the same dosage with *ApoE^-/-^* mice for 8 weeks (Figure S2A). The results showed that Neu5Ac could not increase the plasma Neu5Ac levels (Figure S2B) or promote atherosclerotic plaque formation as well (Figure S2F-H). Similarly, there were no significant changes in liver and kidney function-related indicators (Figure S2C-D) as well as blood lipid levels (Figure S2E). Together, these data strongly suggested that Neu5Ac aggravated AS progression and could be identified as a risk factor for AS with lipid disorder and vascular inflammation.

### Neu5Ac induced vascular endothelial inflammatory injury and contributed to AS

**Figure 2:**
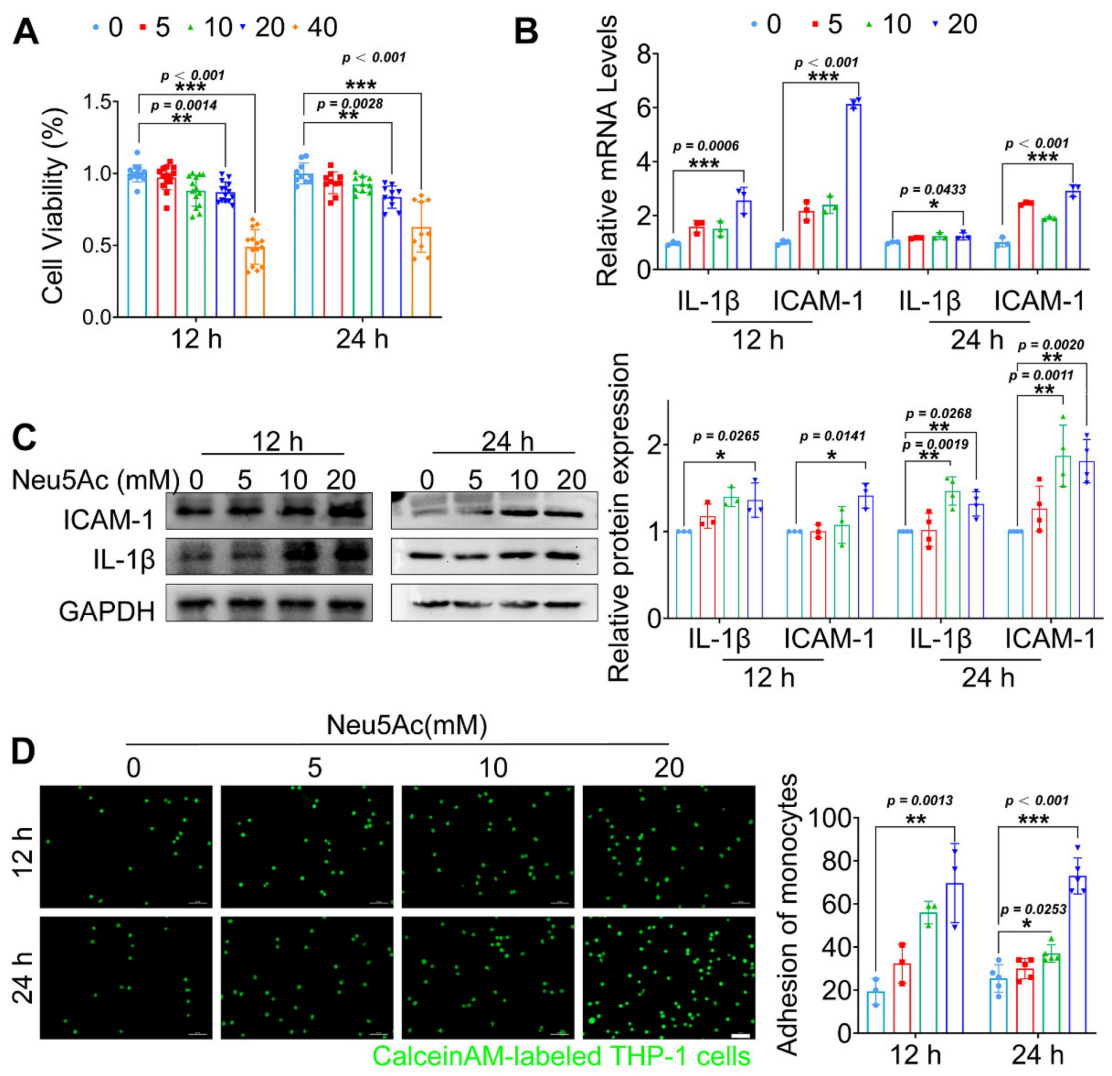
** Neu5Ac induced inflammatory injury in HUVECs.** (A) HUVECs viability was measured by CCK-8, displaying a reduction in cell viability following treatment with Neu5Ac (0, 5, 10, 20, 40 mM) for 12 and 24 h (n > 3 per group). (B) The IL-1β and ICAM-1 mRNA expression were increased in HUVECs after treating with Neu5Ac (0, 5, 10, 20 mM) for 12 and 24 h (n = 3 per group). (C) Western blotting assay showed that Neu5Ac increases IL-1β and ICAM-1 protein levels in a dose and time dependent manner, with quantitative data in the right panel (n ≥ 3 per group). (D) Representative images of monocyte-endothelial adhesion after Neu5Ac (0, 5, 10, 20 mM) treatment for 12 and 24 h in HUVECs, quantification data revealed Neu5Ac increased EC adhesion to monocytes as shown in the right panel (n ≥ 3 per group). Statistical analysis was performed by one-way ANOVA followed by the Tukey test for A-D. HUVEC: human umbilical vein endothelial cells, IL-1β: Interleukin-1β, ICAM-1: Intercellular cell adhesion molecule-1.

Endothelial cells (ECs) activation upon inflammatory cytokines stimulation and then induce monocyte-endothelial adhesion are the initial steps of AS development. Next, we sought to assess whether Neu5Ac promoted AS progress is mediated by disrupting ECs. Owing to the fact that Neu5Ac was metabolized rapidly in body 30, 31, and can act as a ligand for a great variety of molecules or operates as a bio-mask in circulation by linking to glycan chains or proteins 32, the plasma concentrations of Neu5Ac we assayed may not represent the concentrations it actually works. Therefore, we used exaggerated concentrations of Neu5Ac ranged from 0 to 40 mmol/L, the similar experimental strategy with previous study, to investigate its potential role in regulating ECs function 33. We supplemented Neu5Ac to the medium of Human umbilical vein endothelial cells (HUVECs) in order to mimic the elevated Neu5Ac levels *in vitro*. Firstly, we measured the viability of HUVECs after Neu5Ac treatment. As shown in Figure 2A, Neu5Ac significantly reduced the activity of HUVECs in a concentration and time -dependent manner. Accordingly, expression of pro-inflammatory factors such as IL-1β and adhesion molecules ICAM-1were both increased as shown in Figure 2B-C. Furthermore, we measured the role of Neu5Ac on monocyte adhesion to ECs process. Of note, we observed a significant increasing ability of monocyte adhesion to ECs with a concentration and time-dependent manner after Neu5Ac treatment (Figure 2D).

Furthermore, to better understand whether ECs dysfunction is strongly associated with Neu5Ac accumulation, we used tumor necrosis factor-α (TNF-α) stimulating HUVECs to generate a generally inflammatory model. We found that TNF-α induced a visible increasing of Neu5Ac levels in culture media (Figure S3A-B). Azaserine (HBP pathway inhibitor) and Oseltamivir (Neuraminidase inhibitor) are two metabolic inhibitors displaying inhibition on HBP pathway to suppress glucose metabolism transfer into Neu5Ac metabolism pathway and Neu5Ac cleavage into culture media as well. Following inhibition of the Neu5Ac levels in culture HUVECs, we noticed the induction of ICAM-1, VCAM-1, IL-1β expression (Figure S3C), and attenuated monocyte adhesion levels as shown in Figure S3D-E. Together, these data demonstrated that level of metabolite Neu5Ac could be interfered upon ECs dysfunction, and feedback induced vascular endothelial inflammatory injury, which may participate in initiation of AS.

### Neu5Ac induced vascular endothelial injury by promoting ferroptosis pathway

**Figure 3:**
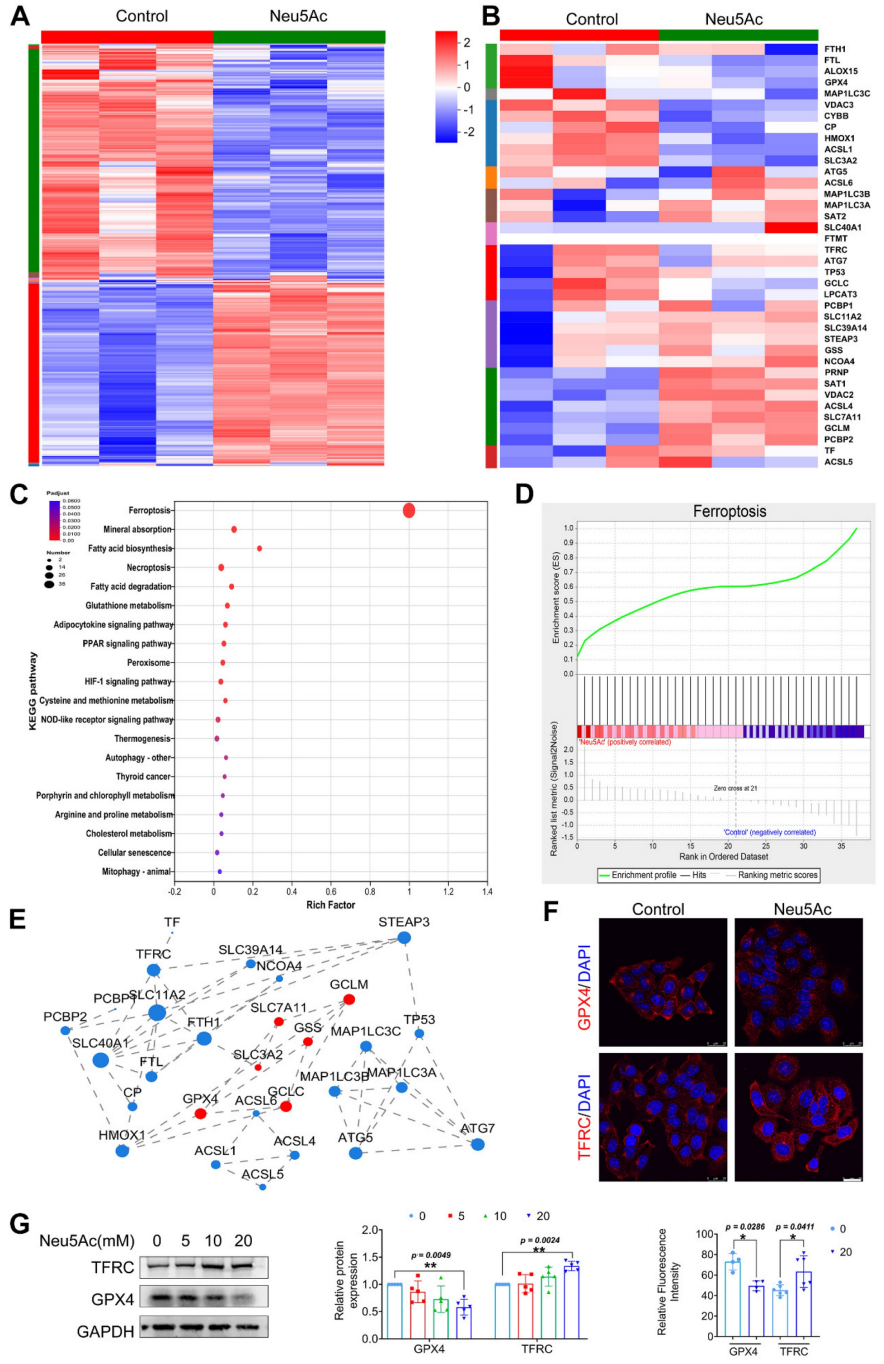
** Neu5Ac induces HUVECs injury by promoting ferroptosis pathway.** HUVECs were treated with Neu5Ac (20 mM) for 24 h. (A) The hierarchically clustered heatmap showed the differentially expressed genes in HUVECs (n = 3 per group). (B) The hierarchically clustered heatmap showed the ferroptosis-related genes in HUVECs (n = 3 per group). (C) Bubbled blot showed the most significantly altered KEGG pathways in HUVECs, and ferroptosis pathway was enriched mostly (n = 3 per group). (D) GSEA analyzed the enrichment plot of the ferroptosis pathway in HUVECs (n = 3 per group). (E) Construction of protein-protein interaction regulatory network based on differentially expressed genes (n = 3 per group). (F) Immunofluorescence staining of GPX4 and TFRC in HUVECs treated with Neu5Ac (20 mM) for 24 h, showing decrease GPX4 level and increase TFRC level in HUVECs exposed Neu5Ac compared with control. Scale bar = 25 μm. The quantification data were shown below (n > 3 per group). (G) HUVECs were treated with Neu5Ac (0, 5, 10, 20 mM) for 24h, and increased TFRC expression, but lows GPX4 expression, and the quantification of these proteins' expression was shown in the right panel (n = 4 per group). Statistical analysis was performed by Student t test for F and one-way ANOVA followed by the Tukey test for G. KEGG: Kyoto Encyclopedia of Genes and Genomes, GSEA: Gene set enrichment analysis, PPI: Protein-Protein Interaction Networks, GPX4: Glutathione peroxidase 4, TFRC: Transferrin receptor.

As reported before, Neu5Ac might be associated with ferroptosis pathway 24, 25. To determine whether ferroptosis pathway is involved in EC injury induced by Neu5Ac, we first performed transcriptome sequencing on ECs upon Neu5Ac treatment. As shown in Figure 3A-B, hierarchically clustered heatmap identified the changes from total genes and found that a series of ferroptosis genes were increased upon Neu5Ac treatment. Kyoto Encyclopedia of Genes and Genomes (KEGG) enrichment analysis also confirmed that ferroptosis pathway significantly altered, and ferroptosis pathway genes significantly enriched in ECs after Neu5Ac treatment analyzing by Gene Set Enrichment Analysis (GSEA) method (Figure 3C-D). The protein-protein interaction (PPI) regulatory network analysis further predicated a high clustering coefficient and potential interaction among ferroptosis pathway genes (Figure 3E). Together, these results indicated that ferroptosis pathway might be activated by Neu5Ac in ECs and then participated in ECs injury. Therefore, we examined the expression of ferroptosis markers, GPX4 and TFRC. The results indicated that expression of GPX4 exhibited decreasing while TFRC expression displayed increasing following Neu5Ac treatment compared with control group in ECs (Figure 3F-G), which further indicated that Neu5Ac activated ferroptosis pathway in ECs.

**Figure 4:**
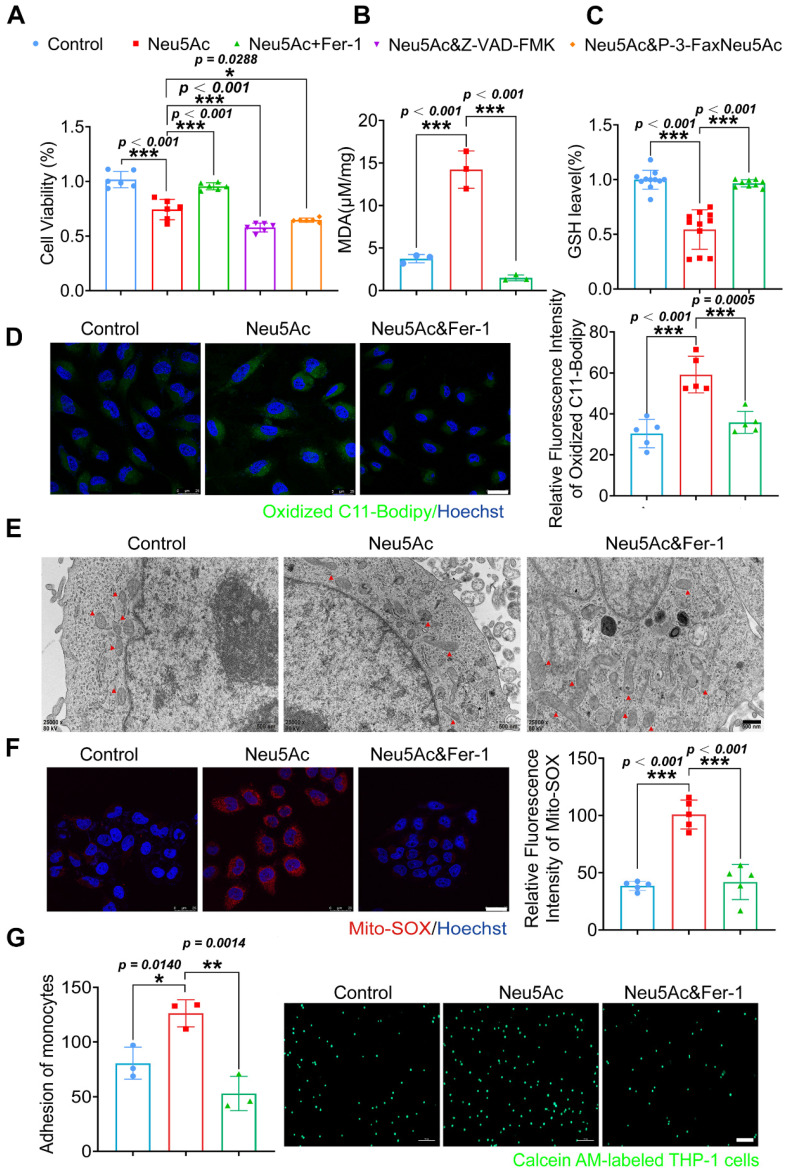
** Fer-1 protected against HUVECs inflammatory injury by abolishing ferroptosis pathway.** (A) CCK-8 assay showed Neu5Ac (20 mM) reduced HUVECs viability, which were reversed by co-treated with Fer-1 (1 μM) rather than Z-VAD-FMK (20 μM) or P-3-Fax-Neu5Ac (100μM) (n > 3 per group). (B) The MDA assay showed Neu5Ac (20 mM) increased MDA levels in HUVECs, which were decreased by co-treated with Fer-1 for 24 h (n = 3 per group). (C) The GSH assay demonstrated Neu5Ac down-regulated GSH levels in HUVECs, which were up-regulated by co-treated with Fer-1 for 24 h (n > 3 per group). (D) Representative images of immunofluorescence staining of C11-BODIPY staining (green) to analyze lipid oxidations. The levels of lipid oxidations were up-regulated in cells with Neu5Ac (20 mM) treatment, which were reversed by Fer-1. The quantification data was shown in the right panel. Scale bar = 25 μm (n = 5 per group). (E) Representative TEM images showed Neu5Ac (20 mM) induced disruption of mitochondrial membrane structure and loss of cristae in HUVECs, which were restored by Fer-1. Scale bar = 500 nm. (F) Immunofluorescence staining of Mito-SOX (red) and Hoechst (blue) in HUVECs, showing increased Mito-SOX levels in HUVECs treated with Neu5Ac (20 mM) and co-treated with Fer-1 can inhibited Mito-SOX. Scale bar = 25 μm (n = 5 per group). (G) Representative images of monocyte-endothelial adhesion. HUVECs were treated with Neu5Ac (20 mM) or Neu5Ac (20 mM) & Fer-1 (1 μM) for 24 h, with quantification data in the right panel (n = 3 per group). Statistical analysis was performed by one-way ANOVA followed by the Tukey test for A-D and F-G. Fer-1: Ferrostatin-1, MDA: Malonaldehyde, GSH: glutathione, TEM: Transmission electron microscope.

Next, we used ferrostatin-1 (Fer-1), a ferroptosis inhibitor, to explore whether anti-ferroptosis drug could abolish the EC injury induced by Neu5Ac. Firstly, we found that Fer-1 could recover the viability of ECs inhibited by Neu5Ac, while apoptosis inhibitor Z-VAD-FMK, or sialyltransferase inhibitor P-3-Fax-Neu5Ac did not counteract Neu5Ac medicated inhibition on ECs (Figure 4A), which indicated that ferroptosis inhibition might be good strategy to abrogate EC injury induced by Neu5Ac. MDA as well as GSH were reported as two common makers of lipid peroxidation, and accumulation of MDA combined with decreasing GSH further trigger ferroptosis 34. In our present study, we also found that Fer-1 alleviated MDA levels and increased GSH levels reduced by Neu5Ac (Figure 4B-C). Moreover, we observed that Neu5Ac increased the level of lipid peroxidation as well as intracellular Fe^2+^ levels by using oxidized C11 BODIPY probe and Ferro Orange probe respectively, while Fer-1 reserved the effect as evidenced by immunofluorescent staining (Figure 4D and Figure S4B). In addition, Fer-1 also down-regulated the expressions of endothelial adhesion molecules and proinflammatory cytokines as well as inhibited monocyte adhesion to ECs induced by Neu5Ac (Figure 4G and Figure S4C). Thus, these results further confirmed that Neu5Ac contributed to lipid peroxidation accumulation and then activated ferroptosis pathway, which was responsible for ECs inflammatory injury.

Mitochondrial fragmentation and cristae enlargement is also typical morphological changes of ECs upon ferroptosis activation, which could release ROS and then promote lipid peroxidation 35. We hypothesized that Neu5Ac might induce mitochondrial dysfunction and then induce ECs ferroptosis. To validate this hypothesis, we investigated mitochondrial morphological changes by using Transmission electron microscopy (TEM) assay. As shown in Figure 4E, Neu5Ac drives mitochondrial atrophy with the disappearance of the bilayer membrane structure and partial loss of the mitochondrial cristae, while Fer-1 counteracted mitochondrial dysfunction mediated by Neu5Ac. Consistent with the above results, we also observed that Fer-1 abolished the increasing of mitochondrial superoxide levels as well as decreasing of mitochondrial membrane potential induced by Neu5Ac (Figure 4F and Figure S4A). Together, these results suggested that Neu5Ac partly induced mitochondrial dysfunction and then facilitated ECs ferroptosis, Fer-1 may develop as a promising therapeutic strategy for Neu5Ac associated AS.

### Fer-1 suppressed AS progression induced by Neu5Ac in* ApoE^-/-^* mice

**Figure 5:**
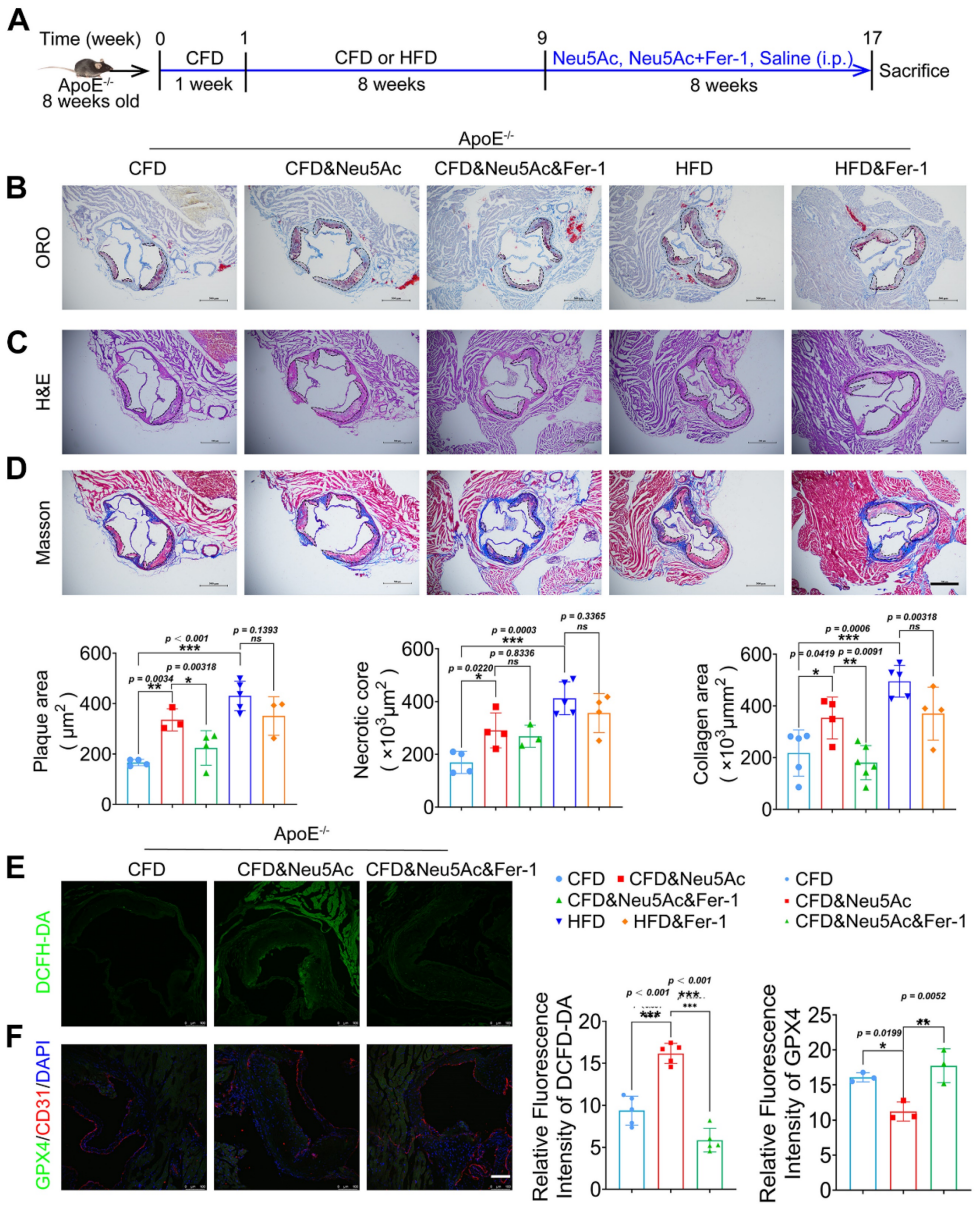
** Fer-1 suppressed Neu5Ac associated-AS progression in ApoE^-/-^ mice.** (A) Experimental design of *ApoE^-/-^* mice combined with Fer-1 treatment. *ApoE^-/-^* mice fed with CFD or HFD and treated intraperitoneally with Neu5Ac (60 mg/kg/d), Neu5Ac&Fer-1 (10 mg/kg/d), Fer-1 (10 mg/kg/d) or saline (60 mg/kg/d) for 8 weeks. (B) Representative microphotograph of Oil Red O-stained aortic sinus. The quantification of Oil Red O positive plaque area was greater in Neu5Ac-treatment group than Neu5Ac&Fer-1 group. Scale bars = 500 μm (n > 3 per group). (C) Representative microphotograph of H&E-stained aortic sinus. The quantification of necrotic core was up-regulated in Neu5Ac-treatment group compared with Neu5Ac&Fer-1 group. Scale bars = 500 μm (n > 3 per group). (D) Representative microphotograph of the aortic sinus stained with Masson's trichrome stain. The quantification of collagen arear was larger in Neu5Ac-treatment groups than Neu5Ac&Fer-1 group. Scale bars = 500 μm (n > 3 per group). (E) Staining of DCFH-DA-positive (green) arear in the aortic sinus from *ApoE^-/-^* mice, showing decreased immunofluorescence intensity of DCFH-DA in the Neu5Ac&Fer-1 group compare with Neu5Ac-treatment group. The quantitative data were shown in the right panel. Scale bar = 25 μm. (n = 4 per group). (F) Representative images of immunofluorescence staining of GPX4 (green), CD31 (red) and DAPI (blue). The quantification of the GPX4 immunofluorescence intensity was increased in the Neu5Ac&Fer-1 group compared with Neu5Ac- treatment group. The quantitative data were shown in the right panel. Scale bar = 25 μm. (n =3 per group). Statistical analysis was performed by one-way ANOVA followed by Tukey test for B-F.

To verify whether Fer-1 could alleviate AS progression induced by Neu5Ac as well as HFD, we established the AS model and then intraperitoneal administration of Fer-1 as shown in Figure 5A. After 8 weeks of treatment, we found a significant reduction of atherosclerotic lesions in *ApoE^-/-^* mice treated with Neu5Ac combined with Fer-1 compared to mice with Neu5Ac-treatment alone (Figure 5B-D). Also, we noticed that Fer-1 showed potential inhibition on plaque progression in HFD-fed mice but with no significant difference. In addition, we measured Neu5Ac levels in plasma from *ApoE^-/-^* mice in different groups. The results showed that Fer-1 could reduce Neu5Ac levels in Neu5Ac treatment group but displayed no significant difference in HFD group (Figure S5A). Biochemical analysis of lipid levels also confirmed that Fer-1 inhibited the TC, LDL levels induced by Neu5Ac or HFD, but exhibited no significant difference in TG and HDL levels disturbed by Neu5Ac or HFD (Figure S5B). H&E staining further showed that Fer-1 failed to reverse the liver as well as kidney morphological changes induced by Neu5Ac or HFD (Figure S5E-F). However, biochemical analysis showed that Fer-1 inhibited the increasing of ALT levels but with less affection on AST or kidney indicators levels (Figure S5C-D). Together, these results indicated that Fer-1 could suppress AS progression especially induced by Neu5Ac.

Furthermore, we examined the indicator for characterizing lipid peroxidation and ferroptosis *in vivo*. Immunofluorescence staining assay from aortic sinus showed that Neu5Ac could increase the levels of lipid peroxidation maker ROS, and down-regulate the expression of ferroptosis maker GPX4, which was consistent with the results *in vitro*. While Fer-1 could significantly inhibit ROS levels and up-regulate GPX4 expression in plaque areas compared with Neu5Ac group (Figure 5E-F). Together, these data further indicated that Neu5Ac promoted ferroptosis pathway and then aggravated AS progression. Fer-1 could counteract ferroptosis activation and displayed anti-atherosclerosis both *in vitro* and *in vivo*.

### Neu5Ac induced SLC3A2 degradation and promoted ferroptosis pathway

**Figure 6:**
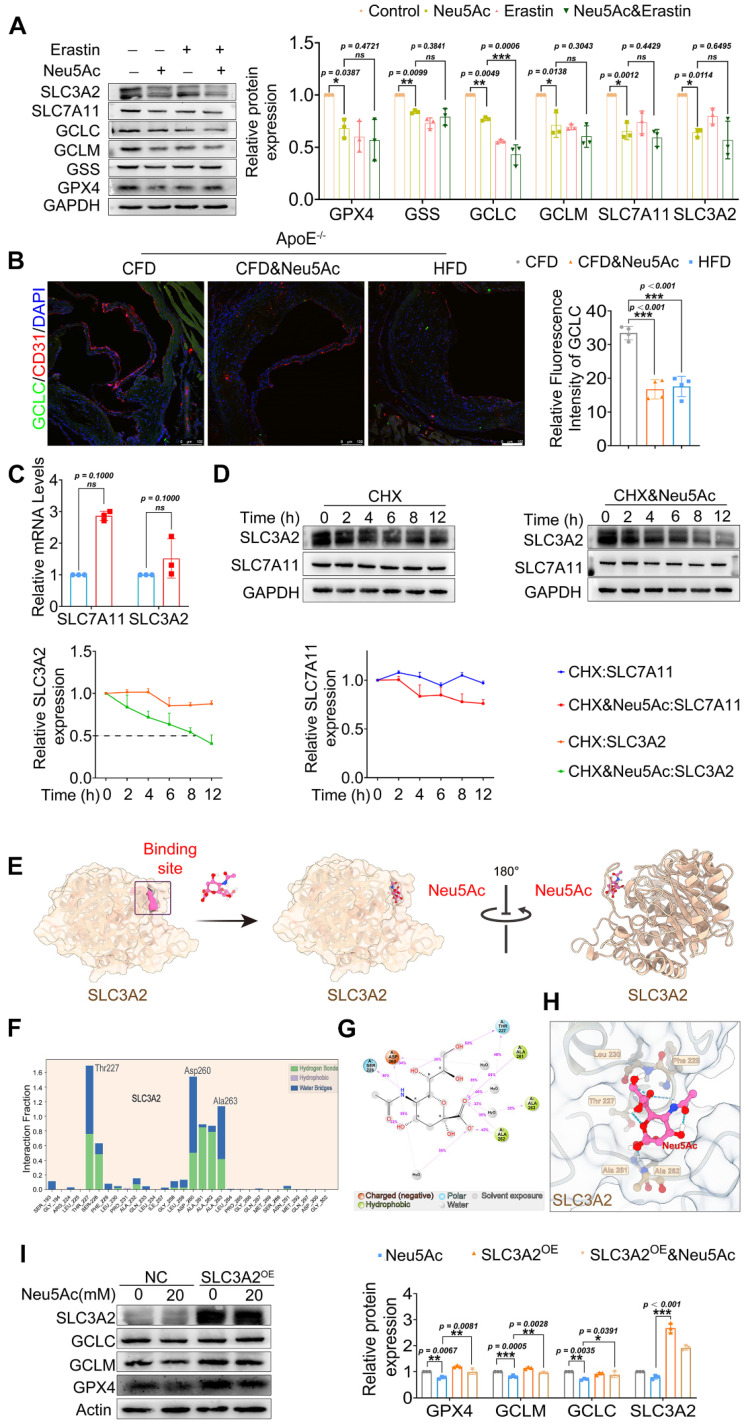
** Neu5Ac induced SLC3A2 degradation and promoted ferroptosis pathway.** (A) Western blotting showed Neu5Ac (20 mM)-induced decreasing of GPX4, GSS, GCLC, GCLM, SLC7A11 and SLC3A2 protein levels. Following co-treatment with Erastin (10 μM), GCLC protein levels were lower than Neu5Ac treated along, with quantitative data in the right panel (n ≥ 3 per group). (B) Representative images of immunofluorescence staining of GCLC (green), CD31 (red), and DAPI (blue) in aortic sinus from *ApoE^-/-^* mice, with quantitative data in the right panel. Scale bar = 100 μm (n = 4 per group). (C) The mRNA levels of SLC7A11 and SLC3A2 were increased after Neu5Ac treatment for 24 h (n = 3 per group). (D) CHX assay was conducted and confirmed Neu5Ac accelerated SLC3A2 protein degradation (n = 3 per group). (E) Representative structure of SLC3A2-Neu5Ac complex modeled with AlphaFold2 algorithm followed by molecular docking. (F-H) Amino acids at the binding site of SLC3A2 and Neu5Ac. (I) HEK293 cells were transfected with SLC3A2 for 48 h, then treated with Neu5Ac (20 mM) for 24 h. The expressions of GPX4, GCLC, GCLM were determined by Western blot, with quantitative data in the right panel (n = 3 per group). Statistical analysis was performed by one-way ANOVA followed by Tukey test for A, B, D, I, and Student t test for the C. CHX: Cycloheximide.

It is widely acknowledged that inactivation of X_C_^-^/GSH/GPX4 system leads to lipid hydroperoxide accumulation and eventually promotes ferroptosis, inducers such as Erastin and RSL3 are shown to inhibit the X_C_^-^/GSH/GPX4 system and then induce ferroptosis activation 36, 37. In our present study, we noticed that a significant reduction of GPX4 level was accompanied with a reduction of intracellular GSH level, which tentatively indicated that Neu5Ac may inhibit X_C_^-^/GSH/GPX4 system in ECs. To investigate whether Neu5Ac-induced ferroptosis is mediated by suppressing X_C_^-^/GSH/GPX4 system, we then investigated the expression of X_C_^-^/GSH/GPX4 system-associated proteins such as SLC7A11, SLC3A2, GPX4, GCLC, GCLM, and GSS, and Erastin was used as a positive control. As shown in Figure 6A, we found that expressions of above proteins were significantly down-regulated upon Neu5Ac treatment, and GCLC displayed further inhibition along with Erastin. Immunofluorescence staining assay also showed that the expression of GCLC in aortic sinus was inhibited in Neu5Ac or HFD-fed *ApoE^-/-^* mice group compared with mice in CFD-fed group (Figure 6B), suggesting that Neu5Ac promoted ferroptosis pathway partly through X_C_^-^/GSH/GPX4 system inhibition.

SLC7A11 as well as SLC3A2 were important transport proteins, always binding together and recognized as the switch of X_C_^-^/GSH/GPX4 system 38. We have found a decreasing protein levels of such transports upon Neu5Ac treatment. However, we detected increasing mRNA levels of SLC7A11 and SLC3A2 (Figure 6C), which partly indicated that Neu5Ac may modulate SLC7A11 and SLC3A2 expressions by affecting protein stability. Then we applied Cycloheximide (CHX), a protease degradation inhibitor, to investigate whether such proteins stability was affected in ECs after Neu5Ac treatment. The results showed that Neu5Ac significantly shortened the half-life of SLC3A2 compared with CHX group, with no obvious effect on half time of SLC7A11 (Figure 6D). These data indicated that SLC3A2 protein degradation maybe partly involved in the inactivation of X_C_^-^/GSH/GPX4 system induced by Neu5Ac. Studies have already reported that Neu5Ac has ability to bind proteins to perform biological functions 39. Therefore, we speculated that Neu5Ac might bind to SLC3A2 and thus regulate its subsequent protein degradation. To verify this speculation, we employed molecular docking techniques to modulated SLC3A2 binding to Neu5Ac. The AlphaFold modelling results showed that Neu5Ac could attach to SLC3A2 and exhibited stable binding ability (Figure 6E). Molecular dynamics optimization also revealed that Neu5Ac could stably bind to the flexible region of SLC3A2 (Figure S7A-D). In addition, results from interaction region between Neu5Ac and SLC3A2 revealed that Neu5Ac mainly bind to SLC3A2 through amino acid residue such as Thr227, Asp260, and Ala263 (Figure 6F-H). Together, these results suggested that Neu5Ac might promote the degradation of SLC3A2 through binding such residue. To further verify the important role of SLC3A2 in ferroptosis activation mediated by Neu5Ac, we used adenoviruses efficiently over-express SLC3A2 and detect the protein levels in regulating ferroptosis pathway. The results showed that SLC3A2 over-expression (SLC3A2^oe^) escalated the X_C_^-^/GSH/GPX4 system proteins compared with the Neu5Ac treatment group (Figure 6I), suggesting that SLC3A2 was critical for ferroptosis activation and Neu5Ac might bind to SLC3A2 and then induced SLC3A2 degradation.

**Figure 7:**
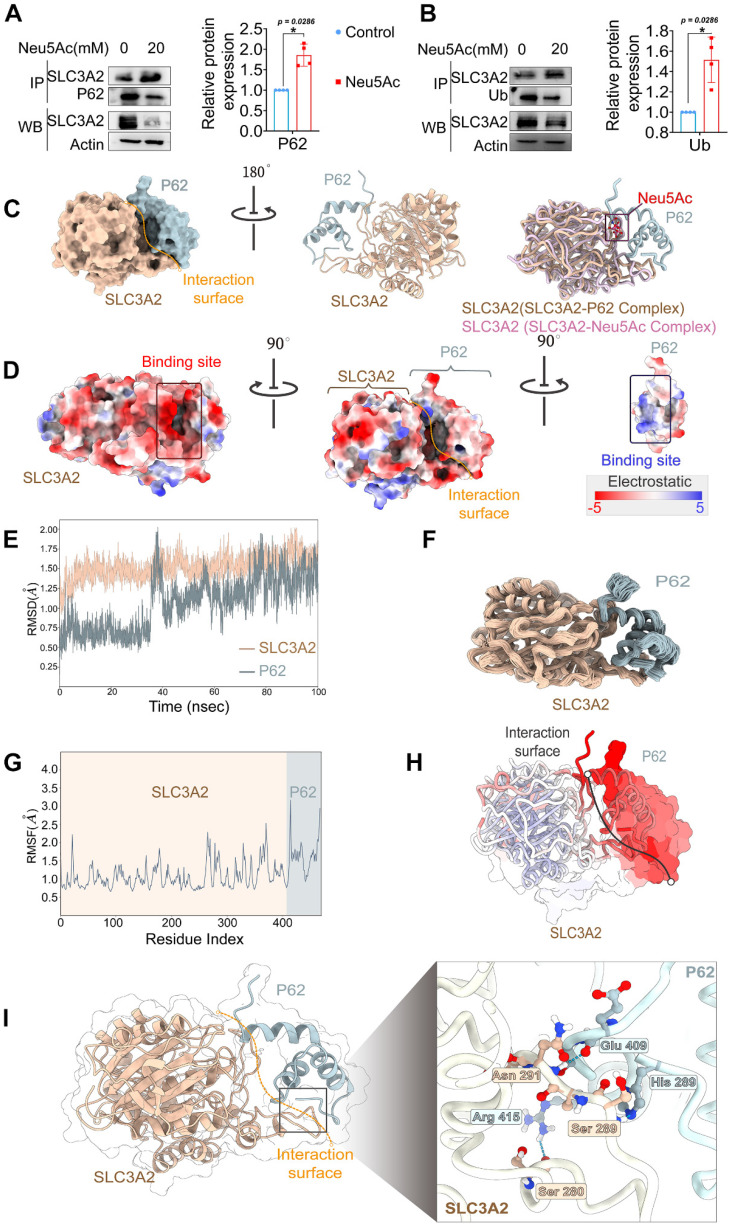
** SLC3A2 could bind P62 in the absence of Neu5Ac treatment.** (A) Co-immunoprecipitation of SLC3A2 and P62 in HUVECs treated with Neu5Ac (20 mM) for 24 h, and the quantification of SLC3A2 protein expression in the precipitant was shown at the bottom (n = 4 per group). (B) Co-immunoprecipitation of SLC3A2 and Ub in HUVECs treated with Neu5Ac (20 mM) for 24 h, and the quantification of SLC3A2 protein expression in the precipitant was shown at the bottom (n = 4 per group). (C) Representative structure of SLC3A2-P62 complex modeled with AlphaFold2 algorithm followed by molecular docking. (D) The space-filling electrostatic surface charge distribution shows a positively charged groove on the SLC3A2 bound to the negatively charged P62. (E-F) RMSD analysis of SLC3A2, and P62 during 100-ns MD simulations. (G-H) RMSF analysis of SLC3A2, and P62. (I) Hydrogen bonds formed by SLC3A2 and P62 complexes during molecular dynamics simulations. Statistical analysis was performed by Student t test for the A, B. P62: Sequestosome 1, Ub: Ubiquitin, RMSD: Root Mean Square Deviation, RMSF: Root Mean Square Fluctuation.

A previous study has reported that SLC3A2 has multiple ubiquitination binding sites, helping it traffick to degradation in a ubiquitin-dependent manner 40. Hence, we speculated that SLC3A2 protein degradation depends on its ubiquitination modifications. To verify this hypothesis, we used co-immunoprecipitation to detect the interaction between SLC3A2 and ubiquitin in HUVECs after Neu5Ac treatment. The results showed that Neu5Ac up-regulated the ubiquitination level of SLC3A2 (Figure 7A), suggesting that SLC3A2 ubiquitination might be involved in its degradation process. P62, serving as a link between ubiquitin and protein degradation, connects the ubiquitin-proteasome system (UPS) to autophagy 41. Our previous study also found that Neu5Ac could induced P62 ubiquitination degradation (data not shown), which indicating that P62 might involve in SLC3A2 degradation process upon Neu5Ac stimulation. We further detected the interaction between SLC3A2 and P62 by using co-immunoprecipitation method. The results showed that SLC3A2 could bind P62, and the binding ability was enhanced after Neu5Ac treatment (Figure 7B). Together, these results indicated that SLC3A2 degradation was probably associated with its ubiquitination and that P62 might regulated SLC3A2 degradation pathway.

**Figure 8:**
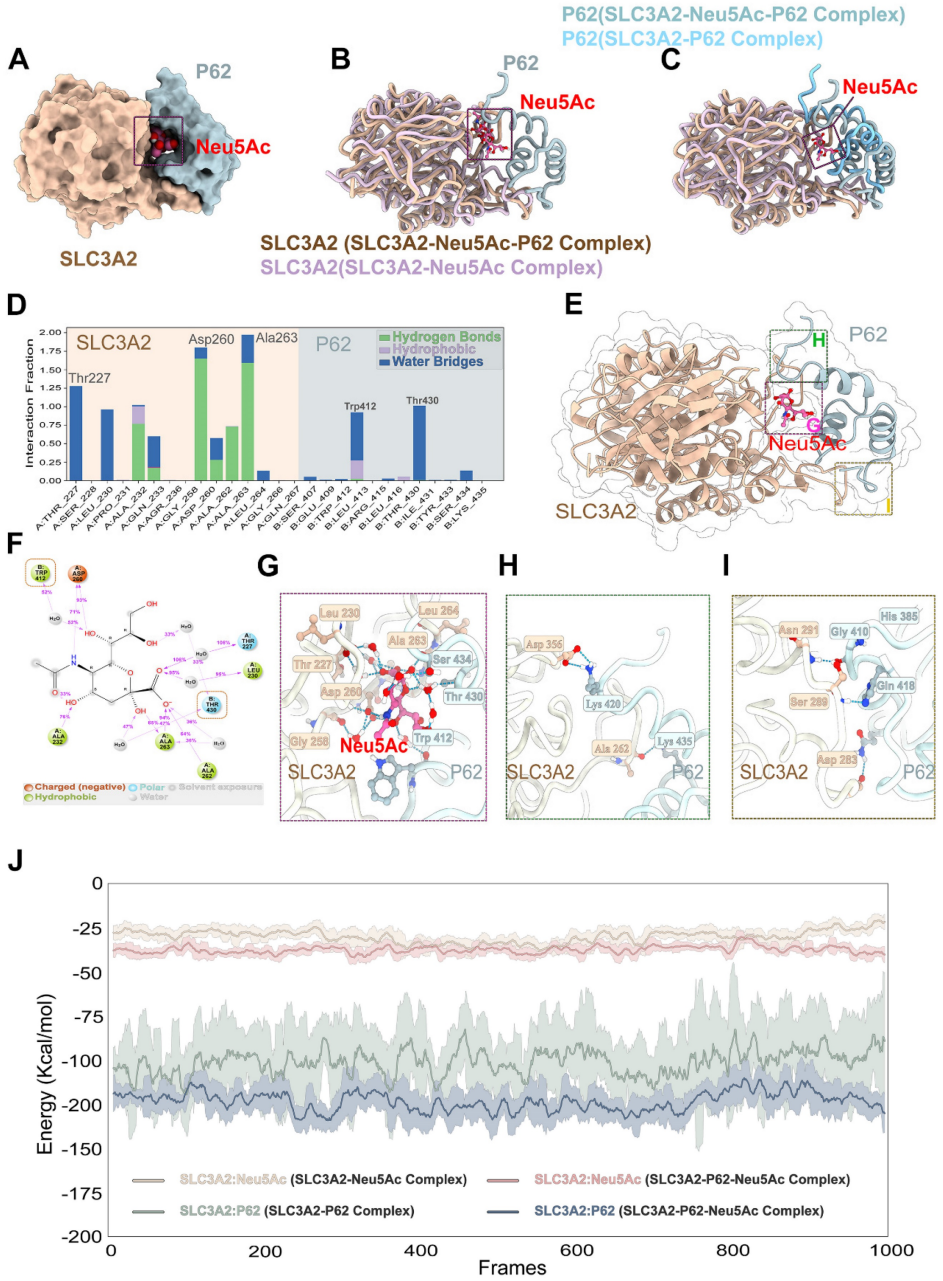
** Neu5Ac promoted SLC3A2 binding to P62.** (A-C) Representative structure of SLC3A2-P62-Neu5Ac complex modeled with AlphaFold2 algorithm followed by molecular docking. (D-G) In the SLC3A2-Neu5Ac-P62 ternary complex, Neu5Ac forms hydrogen bonds with SLC3A2 and P62, respectively, during molecular dynamics simulations. (H-I) Hydrogen bonds formed between SLC3A2 and P62 during molecular dynamics simulations in the SLC3A2-Neu5Ac-P62 ternary complex. (J) Extract the last 5 ns from the trajectories of the molecular dynamics of the SLC3A2-Neu5Ac complex, the SLC3A2-P62 complex and the SLC3A2-Neu5Ac-P62 complex (1000 frames in total) and calculate the free energy of binding between SLC3A2-Neu5Ac complex, the SLC3A2-P62 complex and the SLC3A2-Neu5Ac-P62 for each frame.

To further clarify the relationship between Neu5Ac, SLC3A2, and P62, we applied the molecular docking technique, and the results showed that SLC3A2 binds to P62 stably in the geometry, and Neu5Ac could embedded on the interaction surface of SLC3A2 and P62 (Figure 7C). Also, we found that the positively charged groove of SLC3A2's binding site was geometrically and electronically optimal for binding to P62 with negatively charged surface (Figure 7D). Furthermore, molecular dynamics optimization as well as amino acid hydrogen binding interactions analysis indicated that SLC3A2 connected to P62 through a network of amino acid such as As291-Glu409, Ser289-His289, Ser-280-Arg415 (Figure 7E-I). Overall, these results suggested that SLC3A2 could also bind to P62 in the absence of Neu5Ac treatment. However, the results shown in Figure 8A-C indicated that Neu5Ac could lead to deflections and changes in the structures of SLC3A2 and P62, which making the complex of SLC3A2-Neu5Ac-P62 more stable. Furthermore, molecular dynamics optimization also confirmed the above conclusion (Figure S8A-D).

Further analysis of SLC3A2-Neu5Ac-P62 complex binding site indicated that Neu5Ac could connect SLC3A2 and P62 through series of hydrogen bonds formed by amino acids (Figure 8E-G). In addition, Neu5Ac promoted the interaction of SLC3A2 with P62 amino acid hydrogen bonds (Figure 8H-I). Free energy analysis showed that the average free energy of the SLC3A2-Neu5Ac-P62 complex was lower than that of SLC3A2-P62 complex or SLC3A2-Neu5Ac complex, which suggesting that Neu5Ac enhances SLC3A2 binding to P62 (Figure 8J). Collectively, these results indicated that Neu5Ac increased binding probability of SLC3A2 to P62, and then promoted P62 medicated ubiquitination degradation, which pointing SLC3A2 as a potential target for regulating Neu5Ac-mediated endothelial injury. However, the specific role of P62 as well as its binding sites with SLC3A2, needs to be further explored.

## Discussion

Recent years, targeting metabolism disorder was proposed as a reasonable measure to protect against AS progression. Specially, targeting EC metabolism-associated dysfunction seems to be good strategy to eliminate recruitment of inflammatory cells to injury sites and delay AS plaques buildup 42. In the present study, we mainly focused on metabolite Neu5Ac and confirmed that the levels of Neu5Ac in hyperlipidemia patients as well as *ApoE^-/-^* mice were both up-regulated and displayed as an important risk factor in circulation for EC injury and AS progression. Mechanistic studies revealed that Neu5Ac induced SLC3A2 degradation and then activated ferroptosis pathway, which further promoted ECs injury and ultimately accelerated atherosclerotic process. Additionally, mitochondrial dysfunction also participated in ferroptosis pathway induced by Neu5Ac (Figure 4). These novel findings suggested that inhibition of Neu5Ac levels or ferroptosis in ECs such as Fer-1 may represent an effective anti-inflammatory strategy for treating AS.

N-acetylneuraminic acid (Neu5Ac) is the most abundant sialic acid and together with its substrate N-glycolylneuraminic acid (Neu5Gc) both regulate the vascular homeostasis as reported before 43, 44. It is recognized that Neu5Gc mainly from dietary sources could exacerbate AS progression in *CMAH^-/-^LDLR^-/-^* mice model, a functional lack of Neu5Gc and imitate human-like mice model 44. Kawanishi et al also confirmed that interventional Neu5Ac feeding could mitigate or prevent the Neu5Gc-mediated AS in *CMAH^-/-^LDLR^-/-^* mice model, and has an intrinsic protective effect even in the absence of Neu5Gc feeding 45. However, Soulillou et al commented that *CMAH^-/-^* mice unlikely displayed a universal phenotype that could be extended to humans, and functional lack of LDL receptor predisposes mice to obesity and obesity-associated accelerated AS 46, which indicated that the proposed *CMAH^-/-^LDLR^-/-^* mice model cannot directly reflect the regulation of circulated Neu5Ac on AS progression relating to lipid metabolism disorder and inflammation. In the present study, we also analyzed the differences in CMAH expression values between *ApoE^-/-^* and wild-type mice by using GEO database. The results as shown in Figure S6 indicated that CMAH expression values at different stages of AS displayed no significant changes in tissues and cells from wild-type mice and *ApoE^-/-^* mice, a widely used AS model with lipid disorder and inflammation, which suggesting that the such AS progression might not be associated with the CMAH gene disorder. Also, the above results indicated that effect of Neu5Ac might be differ from *ApoE^-/-^* mice and *CMAH^-/-^LDLR^-/-^* mice models.

Here, we applied *ApoE^-/-^* mice to mimic the accumulation of Neu5Ac in circulation which is similar with HFD-induced AS model, and then we detected the effect of elevated Neu5Ac on vascular homeostasis as well as AS progression. The results indicated that *ApoE^ -/-^* mice were more susceptible to Western diet or Neu5Ac and consequently developed AS. However, *C57BL/6J* mice were less sensitive to Neu5Ac treatment. This may be due to differences in lipid metabolism between two mice models that *ApoE^-/-^* mice was prone to dramatically elevate plasma total cholesterol levels 47, vascular dysfunction and immune disorders compared to wild-type mice 48. Neu5Ac was reported to be highly associated with AS factors like aberrant sialylation of lipoprotein, immune responses 16, also induction of ECs injury obtained from our present study, which may partly promote such atherosclerotic changes. In addition, we also found that inhibition of Neu5Ac production or release to circulation displayed decreasing Neu5Ac levels and down-regulating inflammatory factors, which further confirmed the conclusion that metabolite Neu5Ac itself promoted arterial disease progression both *in vivo* and *in vitro* and also expand our previous research 49. However, there are some researchers who found that exogenous Neu5Ac supplementation with water or food displayed opposite effect on AS progression in* ApoE^-/-^* mice 45, 50, 51. Hou et al. showed that exogenous Neu5Ac supplementation via dietary water improved the reserve cholesterol transport in *ApoE^-/-^* mice, which allowed peripheral cells to excrete excess cholesterol and exert anti-AS action 52. Similarly, Zhang et al. found that feeding rats free Neu5Ac by gavage suppressed HFD-induced hyperlipidemia 53, 54. The distinct effects of Neu5Ac on *ApoE^-/-^* mice probably related to its administration method, which may induce different concentration of Neu5Ac in circulation, and requires further studies to reveal. But our present research strategy as well as results were in line with the research reported by Zhang et al. that the elevated Neu5Ac in artery could directly injury organs homeostasis where it communicates, and was able to trigger myocardial injury both in rats and in ventricular myocytes 33. Similarly, studies have also shown that elevated circulating Neu5Ac level is associated with accelerated immune exhaustion (CD4^+^ T-cell compartment) and recognition-memory impairment. Suzzid et al. found that subcutaneous injection with a high dose of Neu5Ac into 5xFAD mice could replicate a long-term HFD feeding mice model with elevated plasma levels of Neu5Ac 55, which partly indicated that the strategy we used was reliable to reflect the effect of elevated Neu5Ac on vascular microenvironment as well as AS progression. Also, the present study release signals that Neu5Ac may be a potential inducer of vascular homeostatic imbalance in lipid disorder mice and AS susceptible patients or patients with vascular inflammation, needing to pay extra attention to the intake of food such as bird's nest rich in Neu5Ac 56.

In addition, Neu5Ac could also participate in the regulation of AS process through modulating its own metabolic pathway, which is partly depending on some rate-limiting enzymes during its biosynthetic pathway 57-61. Studies have found that Neu5Ac accumulates intracellularly from two ways: 1) cell uptakes glucose and then undergoes hexosamine pathway to generate N-acetylglucosamine, which then enters into Neu5Ac synthesis pathway; 2) neuraminidase (Neus) family such as Neu1 shears and releases Neu5Ac from the terminal end of sialic acid linkage 62. Li et al. revealed that Neus and plasma Neu5Ac levels were significantly increased in patients with heart failure compared to normal subjects 63. In addition, a previous study reported an up-regulation of Neu1 levels in invading immune cells and locally in cardiomyocytes upon ischemia-reperfusion 64. In our present study, we also found that the Neu5Ac level was decreased upon Oseltamivir and Oseltamivir treatment, two inhibitors which could block Neu5Ac cleavage into culture media and suppress glucose metabolism transfer into Neu5Ac metabolism respectively 65. And TNF-α could induce a visible increase of Neu5Ac levels (25-100 ng/mL) in culture media, probably owing to the induction of NEU1 or GFAT1 as reported before 66, 67. However, we noticed the induction of ICAM-1, VCAM-1, IL-1β expression as well as the attenuated monocyte adhesion levels after inhibition of the Neu5Ac levels in culture HUVECs. These results further indicated that level of metabolite Neu5Ac could be interfered by metabolic enzymes upon ECs dysfunction, by then inducing vascular endothelial inflammatory injury, which may continuously promote the initiation of AS.

Ferroptosis, an iron-dependent programmed cell death, has been widely studied and associated with cardiac pathology 68. Many ferroptosis inhibitors such as ferrostatins and liproxstatins have been confirmed to protect the kidney 69, brain 70, and heart 71. Our experiments revealed for the first time that Neu5Ac activated ferroptosis in vascular endothelial by triggering accumulation of ROS, Fe^2+^, and lipid peroxidation, which are typical features of ferroptosis 21. As mitochondrial plays an essential role in ferroptosis 72, we also noticed that Neu5Ac induces mitochondrial oxidative stress and promotes ferroptosis, supporting the study that mitochondrial oxidative stress induces Fe^2+^ disorder and activated ferroptosis 73. Furthermore, we found that Fer-1 was effective in reducing endothelial cell inflammation, restoring mitochondrial function, and suppressing the atherosclerotic progression, which was also consistent with the previous report 74. Overall, we revealed that Neu5Ac activates ferroptosis to exacerbate endothelial inflammation injury and ultimately accelerates the development of AS. Fer-1, a widely used ferroptosis inhibitor, was reported to present anti-atherosclerosis through attenuating lipid peroxidation and endothelial dysfunction in *ApoE^-/-^* mice 27, which can be also confirmed in our present work.

GSH depletion has been shown to affect GPX4 activity and stability, thus causing cells more sensitive to ferroptosis 75. Previous studies have demonstrated that GPX4 utilizes two molecules of GSH to scavenge lipid hydroperoxides, and was down-regulated during AS progression 76. In the present study, we also noticed that Neu5Ac decreases intracellular GSH levels and inhibits GPX4 expression at the same time. Thus, our data indicated that GSH may serve as the critical factor in modulating the EC injury induced by Neu5Ac. As evidenced showed that intracellular GSH synthesis is mainly regulated by the substrate cysteine and system X_C_^-^ consisting of SLC7A11, SLC3A2, GCLC, and GCLM, which especially play important roles in switching intracellular transport of cysteine. Activation of SLC7A11/ SLC3A2 allows cells to restore redox homeostasis and maintain survival under stressful conditions 77. Conversely, disruption of System X_C_^-^ with Erastin or its analogues leads to glutathione depletion, endoplasmic reticulum stress, and ultimately cell death 78, 79. In line with the previous study, we found that Neu5Ac triggers ECs inflammatory injury partly depending on the inhibition of system X_C_^-^. SLC7A11 is the major functional subunit of system X_C_^-^, and SLC3A2 acts as a chaperone protein to support the function of SLC7A11, which both important for the function of system X_C_^-^ 80. Here, we observed that Neu5Ac up-regulated the mRNA levels of SLC3A2/SLC7A11, but induced degradation of SLC3A2, which may be responsible for the decreasing of SLC3A2/SLC7A11 proteins levels and decreasing of GSH levels, similar to the previous study 81, 82.

It has been reported that SLC3A2 trafficking is ubiquitin dependent and multiple residues within SL3A2 were identified as ubiquitination sites 40. Eyster et al. have reported that both the membrane-associated RING-CH8 E3 ubiquitin ligase and membrane-associated RING-CH1 E3 ubiquitin ligase could lead to the ubiquitination of SLC3A2, and further recognized by the proteasome 83, thus suggesting that SLC3A2 may be degraded through ubiquitin-proteasome system (UPS). However, recent study pointed out that lower expression of SLC3A2 significantly correlates with increased autophagy levels 84. P62 was one of the mostly important elements which devoted in connecting ubiquitinated proteins and autophagic degradation 41, which was a multifunctional protein consisting of several protein-protein interaction domains, such as PB1, ZZ, NLS, NES, LIR, KIR, UBA 85, 86. As reported before, P62 serves as an integration center for multiple functions, such as autophagosome formation, delivery of ubiquitinated proteins to the proteasome, and aggregate formation for autophagic clearance. Recently, numerous studies showed that autophagy dysfunction regulated by P62 was closely related to the AS progress 87-89. Besides, growing evidences have suggested that P62 bound ubiquitin was indicated as a signal for autophagic degradation. Peter et al demonstrated that P62 could recognize Ub substrates, and then bind ubiquitinated proteins to influence disease processes, such as cancer, heart failure and AS 90, which suggesting that P62 could be a good candidate for ubiquitination degradation. Therefore, we presumed that Neu5Ac may induce ubiquitination of SLC3A2, and subsequently degradation by P62 activation pathway. Based on the data obtained from co-immunoprecipitation, we observed that Neu5Ac increased the interaction between SLC3A2 with ubiquitin as well as P62, suggesting Neu5Ac up-regulated SLC3A2 ubiquitination level. However, whether ubiquitinated SLC3A2 will specifically bind to P62 and then degraded still unclear. Thus, we applied AlphaFold modeling and ZDOCK, followed by molecular dynamics (MD) optimization. The results showed that Neu5Ac could enhance the binding of SLC3A2 with P62. Together, these evidences suggested a critical role of P62 in binding with SLC3A2, and also partly confirmed our previous hypothesis. However, the deep mechanism needs further investigation in the future.

Although we have obtained evidences that elevated circulating Neu5Ac could promote AS procession through SLC3A2 medicated ferroptosis pathway, there still presents some limitations in our study. Firstly, we used *ApoE^-/-^* mice to mimic human AS patients. However, it could not completely reflect the effect of Neu5Ac on AS progression. Some other models like *LDLR^-/-^* mice should be included to investigate its potential function on different pathogenic background. Secondly, we applied intraperitoneal injection strategy to achieve high levels of Neu5Ac in plasma, more methods such as oral, intravenous or intraperitoneal supplementation are needed to confirm the role of Neu5Ac* in vivo*. Also, the detection of Neu5Ac levels at different stage of AS were necessary, which may account for different functions of Neu5Ac *in vivo*. Thirdly, the specific manner of SLC3A2 degradation, especially its connection with P62 upon Neu5Ac stimulation, needs to be further demonstrated.

Taken together, the results present here showed that Neu5Ac served as a risk factor for those patients with vascular inflammation or susceptibility to AS. Moreover, we found that Neu5Ac induced EC ferroptosis and exacerbated AS progression through SLC3A2 pathway. Therapeutic strategies for inhibiting ferroptosis such as Fer-1 may have therapeutic potential against AS progression.

## Supplementary Material

Supplementary figures.

## Figures and Tables

**Figure 9:**
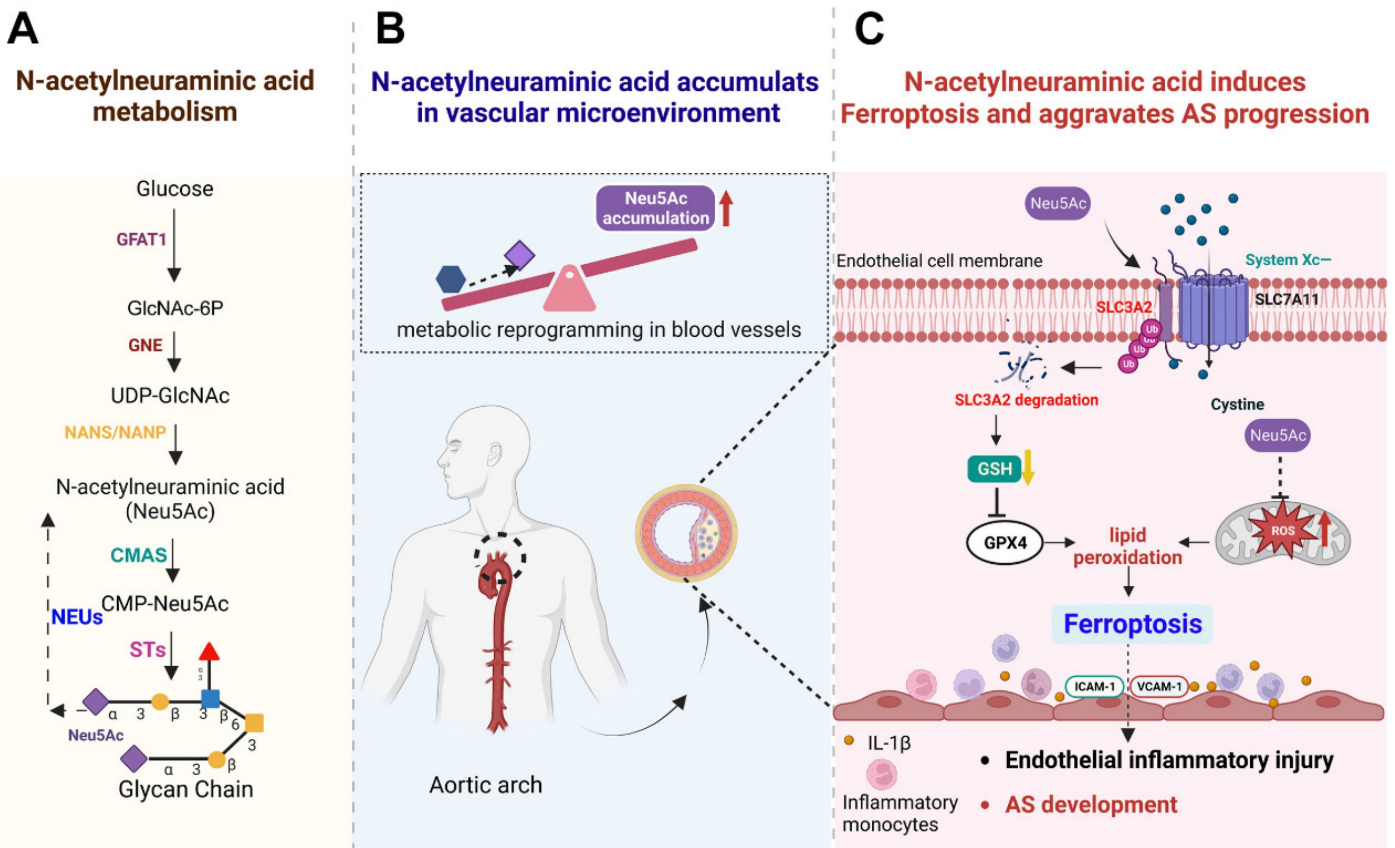
** Schematic concept of metabolite Neu5Ac in inducing ferroptosis and aggravating AS progression.** (A) Normal Neu5Ac metabolism pathway in humans with several important enzymes and its catalytic substrate; (B) Upon metabolism disorder such as glucose disorder, Neu5Ac accumulates in vascular microenvironment and identify as a characteristic biomarker of AS progression; (C) Evaluated Neu5Ac in circulation promotes SLC3A2 degradation and decreases GSH levels, further induces lipid peroxides accumulation and activates EC ferroptosis. Meanwhile, Neu5Ac induces mitochondrial damage and releases ROS, which further exacerbates ferroptosis. Together, Neu5Ac induces EC injury and secretes a variety of inflammatory factors which capture circulating monocytes and adhere to the vascular endothelium, leading to the AS progression. Fer-1 could inhibit lipid peroxides and then attenuate ECs injury as well as protect against premature AS induced by Neu5Ac accumulation. GFAT1: Fructose-6-phosphate aminotransferase; GlcNAc-6P: GlcNAc-6-phosphate; GNE: hydrolyzing UDP-GlcNAc 2-epimerase/ManNAc-6-kinase; NANS: Neu5Ac-9-P synthetase; NANP: Neu5Ac-9-P phosphatase; CMAS: cytidine monophosphate N-acetylneuraminic acid synthetase; STs: sialyltransferases; NEUs: neuraminidases.

## Abbreviations

ALT: Alanine aminotransferase
AST: Aspartate aminotransferase
AZA: Azaserine
CCK-8: Cell Count Kit 8
CFD: Controlled-fat diet
CHO: Cholesterol
CHX: Cycloheximide
CVD: Cardiovascular disease
Fe^2+^: Ferrous iron
GPX4: Glutathione Peroxidase 4
GSH: Glutathione
HDL: High-density lipoprotein
HE: Hematoxylin-Eosin
HFD: High-fat diet
HUVECs: Human umbilical vein endothelial cells
ICAM-1: Intercellular Adhesion Molecule 1
IL-1β: Interleukin-1β
IP: Immunoprecipitation
LDL: Low-density lipoprotein
MAL Ⅰ: Maackia Amurensis Lectin Ⅰ
MDA: Malondialdehyde
Neu5Ac: N-acetylneuraminic acid
ORO: Oil red O
Oselt: Oseltamivir
PFA: Paraformaldehyde
ROS: Reactive oxygen species
SIA: Sialic acid
SLC3A2: Cystine/glutamate anti-porter solute carrier family 3 member 2
SLC7A11: Cystine/glutamate anti-porter solute carrier family 7 member 11
SNA: Sambucus Nigra Lectin
TFRC1: Transferrin receptor 1
TG: Triglycerides
TNF-α: Tumor Necrosis Factor alpha
VECs: Vascular Endothelial cells
WGA: Wheat germ agglutinin

## References

[1] Health effects of dietary risks in 195 countries, 1990-2017 (2019). a systematic analysis for the Global Burden of Disease Study 2017. Lancet.

[2] Benjamin EJ, Muntner P, Alonso A, Bittencourt MS, Callaway CW, Carson AP (2019). Heart Disease and Stroke Statistics-2019 Update: A Report From the American Heart Association. Circulation.

[3] Libby P (2021). The changing landscape of atherosclerosis. Nature.

[4] Liang S, Zhang J, Ning R, Du Z, Liu J, Batibawa JW (2020). The critical role of endothelial function in fine particulate matter-induced atherosclerosis. Part Fibre Toxicol.

[5] Li X, Sun X, Carmeliet P (2019). Hallmarks of Endothelial Cell Metabolism in Health and Disease. Cell Metab.

[6] Xu S, Ilyas I, Little PJ, Li H, Kamato D, Zheng X (2021). Endothelial Dysfunction in Atherosclerotic Cardiovascular Diseases and Beyond: From Mechanism to Pharmacotherapies. Pharmacol Rev.

[7] Rinschen MM, Ivanisevic J, Giera M, Siuzdak G (2019). Identification of bioactive metabolites using activity metabolomics. Nat Rev Mol Cell Biol.

[8] Yang K, Fan M, Wang X, Xu J, Wang Y, Gill PS (2022). Lactate induces vascular permeability via disruption of VE-cadherin in endothelial cells during sepsis. Sci Adv.

[9] Rossi M, Altea-Manzano P, Demicco M, Doglioni G, Bornes L, Fukano M (2022). PHGDH heterogeneity potentiates cancer cell dissemination and metastasis. Nature.

[10] van Karnebeek CDM, Bonafé L, Wen X-Y, Tarailo-Graovac M, Balzano S, Royer-Bertrand B (2016). NANS-mediated synthesis of sialic acid is required for brain and skeletal development. Nat Genet.

[11] Kontou M, Weidemann W, Bork K, Horstkorte R (2009). Beyond glycosylation: sialic acid precursors act as signaling molecules and are involved in cellular control of differentiation of PC12 cells. Biol Chem.

[12] Wu D, Gilormini P-A, Toda S, Biot C, Lion C, Guérardel Y (2022). A novel C-domain-dependent inhibition of the rainbow trout CMP-sialic acid synthetase activity by CMP-deaminoneuraminic acid. Biochem Biophys Res Commun.

[13] Toussaint K, Appert-Collin A, Morjani H, Albrecht C, Sartelet H, Romier-Crouzet B (2022). Neuraminidase-1: A Sialidase Involved in the Development of Cancers and Metabolic Diseases. Cancers.

[14] Guruaribam VD, Sarumathi T (2020). Relevance of serum and salivary sialic acid in oral cancer diagnostics. J Cancer Res Ther.

[15] Sillanaukee P, Pönniö M, Jääskeläinen IP (1999). Occurrence of sialic acids in healthy humans and different disorders. Eur J Clin Invest.

[16] Zhang C, Chen J, Liu Y, Xu D (2019). Sialic acid metabolism as a potential therapeutic target of atherosclerosis. Lipids Health Dis.

[17] White EJ, Gyulay G, Lhoták Š, Szewczyk MM, Chong T, Fuller MT (2018). Sialidase down-regulation reduces non-HDL cholesterol, inhibits leukocyte transmigration, and attenuates atherosclerosis in ApoE knockout mice. J Biol Chem.

[18] Mandic R, Opper C, Krappe J, Wesemann W (2002). Platelet sialic acid as a potential pathogenic factor in coronary heart disease. Thromb Res.

[19] Varki A (2008). Sialic acids in human health and disease. Trends Mol Med.

[20] Zhang J-Y, Chen Q-Q, Li J, Zhang L, Qi L-W (2021). Neuraminidase 1 and its Inhibitors from Chinese Herbal Medicines: An Emerging Role for Cardiovascular Diseases. Am J Chin Med.

[21] Yang WS, SriRamaratnam R, Welsch ME, Shimada K, Skouta R, Viswanathan VS (2014). Regulation of ferroptotic cancer cell death by GPX4. Cell.

[22] Ouyang S, You J, Zhi C, Li P, Lin X, Tan X (2021). Ferroptosis: the potential value target in atherosclerosis. Cell Death Dis.

[23] Cole AL, Subbanagounder G, Mukhopadhyay S, Berliner JA, Vora DK (2003). Oxidized phospholipid-induced endothelial cell/monocyte interaction is mediated by a cAMP-dependent R-Ras/PI3-kinase pathway. Arterioscler Thromb Vasc Biol.

[24] Cavezzi A, Menicagli R, Troiani E, Corrao S (2022). COVID-19, Cation Dysmetabolism, Sialic Acid, CD147, ACE2, Viroporins, Hepcidin and Ferroptosis: A Possible Unifying Hypothesis. F1000Res.

[25] Li M-N, Bao B-W, Si-Yu D, Chun-Fei J, Xiao-Jun S, Da-Sheng G (2022). Correlation between plasma glutathione peroxidase 4 and N-acetylneuraminic acid levels with clinical risk stratification and prognosis of patients with acute coronary syndrome. Saudi Med J.

[26] Marais AD (2021). Apolipoprotein E and Atherosclerosis. Curr Atheroscler Rep.

[27] Bai T, Li M, Liu Y, Qiao Z, Wang Z (2020). Inhibition of ferroptosis alleviates atherosclerosis through attenuating lipid peroxidation and endothelial dysfunction in mouse aortic endothelial cell. Free Radic Biol Med.

[28] Crook MA, Earle K, Morocutti A, Yip J, Viberti G, Pickup JC (1994). Serum sialic acid, a risk factor for cardiovascular disease, is increased in IDDM patients with microalbuminuria and clinical proteinuria. Diabetes Care.

[29] Lindberg G, Eklund GA, Gullberg B, Råstam L (1991). Serum sialic acid concentration and cardiovascular mortality. BMJ.

[30] Nöhle U, Beau JM, Schauer R (1982). Uptake, metabolism and excretion of orally and intravenously administered, double-labeled N-glycoloylneuraminic acid and single-labeled 2-deoxy-2,3-dehydro-N-acetylneuraminic acid in mouse and rat. Eur J Biochem.

[31] Taguchi R, Minami A, Matsuda Y, Takahashi T, Otsubo T, Ikeda K (2015). Preferential Accumulation of 14C-N-Glycolylneuraminic Acid over 14C-N-Acetylneuraminic Acid in the Rat Brain after Tail Vein Injection. PLoS One.

[32] Varki A (2007). Glycan-based interactions involving vertebrate sialic-acid-recognizing proteins. Nature.

[33] Zhang L, Wei T-T, Li Y, Li J, Fan Y, Huang F-Q (2018). Functional Metabolomics Characterizes a Key Role for N-Acetylneuraminic Acid in Coronary Artery Diseases. Circulation.

[34] Xu Y, Li Y, Li J, Chen W (2022). Ethyl carbamate triggers ferroptosis in liver through inhibiting GSH synthesis and suppressing Nrf2 activation. Redox Biol.

[35] Stockwell BR, Jiang X, Gu W (2020). Emerging Mechanisms and Disease Relevance of Ferroptosis. Trends Cell Biol.

[36] Kuang F, Liu J, Tang D, Kang R (2020). Oxidative Damage and Antioxidant Defense in Ferroptosis. Front Cell Dev Biol.

[37] Li F-J, Long H-Z, Zhou Z-W, Luo H-Y, Xu S-G, Gao L-C (2022). System X /GSH/GPX4: An important antioxidant system for the ferroptosis in drug-resistant solid tumor therapy. Front Pharmacol.

[38] Koppula P, Zhuang L, Gan B (2021). Cystine transporter SLC7A11/xCT in cancer: ferroptosis, nutrient dependency, and cancer therapy. Protein Cell.

[39] Vattepu R, Sneed SL, Anthony RM (2022). Sialylation as an Important Regulator of Antibody Function. Front Immunol.

[40] McCracken AN, Edinger AL (2013). Nutrient transporters: the Achilles' heel of anabolism. Trends Endocrinol Metab.

[41] Liu WJ, Ye L, Huang WF, Guo LJ, Xu ZG, Wu HL (2016). p62 links the autophagy pathway and the ubiqutin-proteasome system upon ubiquitinated protein degradation. Cell Mol Biol Lett.

[42] Luo J-Y, Cheng CK, He L, Pu Y, Zhang Y, Lin X (2022). Endothelial UCP2 Is a Mechanosensitive Suppressor of Atherosclerosis. Circ Res.

[43] Samraj AN, Läubli H, Varki N, Varki A (2014). Involvement of a non-human sialic Acid in human cancer. Front Oncol.

[44] Kawanishi K, Dhar C, Do R, Varki N, Gordts PLSM, Varki A (2019). Human species-specific loss of CMP-N-acetylneuraminic acid hydroxylase enhances atherosclerosis via intrinsic and extrinsic mechanisms. Proc Natl Acad Sci U S A.

[45] Kawanishi K, Coker JK, Grunddal KV, Dhar C, Hsiao J, Zengler K (2021). Dietary Neu5Ac Intervention Protects Against Atherosclerosis Associated With Human-Like Neu5Gc Loss-Brief Report. Arterioscler Thromb Vasc Biol.

[46] Soulillou J-P, Cozzi E, Galli C, Bach J-M (2020). Can we extrapolate from a Cmah -/- Ldlr -/- mouse model a susceptibility for atherosclerosis in humans?. Proc Natl Acad Sci U S A.

[47] Emini Veseli B, Perrotta P, De Meyer GRA, Roth L, Van der Donckt C, Martinet W (2017). Animal models of atherosclerosis. Eur J Pharmacol.

[48] Gisterå A, Ketelhuth DFJ, Malin SG, Hansson GK (2022). Animal Models of Atherosclerosis-Supportive Notes and Tricks of the Trade. Circ Res.

[49] Hu X, Li Y, Chen Q, Wang T, Ma L, Zhang W (2023). Sialic acids promote macrophage M1 polarization and atherosclerosis by upregulating ROS and autophagy blockage. Int Immunopharmacol.

[50] Guo S, Tian H, Dong R, Yang N, Zhang Y, Yao S (2016). Exogenous supplement of N-acetylneuraminic acid ameliorates atherosclerosis in apolipoprotein E-deficient mice. Atherosclerosis.

[51] Yida Z, Imam MU, Ismail M, Ismail N, Ideris A, Abdullah MA (2015). High fat diet-induced inflammation and oxidative stress are attenuated by N-acetylneuraminic acid in rats. J Biomed Sci.

[52] Hou P, Hu S, Wang J, Yang Z, Yin J, Zhou G (2019). Exogenous supplement of N-acetylneuraminic acid improves macrophage reverse cholesterol transport in apolipoprotein E-deficient mice. Lipids Health Dis.

[53] Yida Z, Imam MU, Ismail M, Wong W, Abdullah MA, Ideris A (2015). N-Acetylneuraminic acid attenuates hypercoagulation on high fat diet-induced hyperlipidemic rats. Food Nutr Res.

[54] Yida Z, Imam MU, Ismail M, Ismail N, Azmi NH, Wong W (2015). N-Acetylneuraminic Acid Supplementation Prevents High Fat Diet-Induced Insulin Resistance in Rats through Transcriptional and Nontranscriptional Mechanisms. Biomed Res Int.

[55] Suzzi S, Croese T, Ravid A, Gold O, Clark AR, Medina S (2023). N-acetylneuraminic acid links immune exhaustion and accelerated memory deficit in diet-induced obese Alzheimer's disease mouse model. Nat Commun.

[56] Ling AJW, Chang LS, Babji AS, Latip J, Koketsu M, Lim SJ (2022). Review of sialic acid's biochemistry, sources, extraction and functions with special reference to edible bird's nest. Food Chem.

[57] Oetke C, Hinderlich S, Brossmer R, Reutter W, Pawlita M, Keppler OT (2001). Evidence for efficient uptake and incorporation of sialic acid by eukaryotic cells. Eur J Biochem.

[58] Sprenger N, Duncan PI (2012). Sialic acid utilization. Adv Nutr.

[59] Choi SSH, Baldwin N, Wagner VO, Roy S, Rose J, Thorsrud BA (2014). Safety evaluation of the human-identical milk monosaccharide sialic acid (N-acetyl-d-neuraminic acid) in Sprague-Dawley rats. Regul Toxicol Pharmacol.

[60] Morgan BL, Winick M (1980). Effects of administration of N-acetylneuraminic acid (NANA) on brain NANA content and behavior. J Nutr.

[61] De Vries GH, Barondes SH (1971). Incorporation of [14C]N-acetyl neuraminic acid into brain glycoproteins and gangliosides *in vivo*. J Neurochem.

[62] Yang H, Lu L, Chen X (2021). An overview and future prospects of sialic acids. Biotechnol Adv.

[63] Li C, Zhao M, Xiao L, Wei H, Wen Z, Hu D (2021). Prognostic Value of Elevated Levels of Plasma N-Acetylneuraminic Acid in Patients With Heart Failure. Circ Heart Fail.

[64] Heimerl M, Sieve I, Ricke-Hoch M, Erschow S, Battmer K, Scherr M (2020). Neuraminidase-1 promotes heart failure after ischemia/reperfusion injury by affecting cardiomyocytes and invading monocytes/macrophages. Basic Res Cardiol.

[65] Boivin G (2013). Detection and management of antiviral resistance for influenza viruses. Influenza Other Respir Viruses.

[66] Chen G-Y, Brown NK, Wu W, Khedri Z, Yu H, Chen X (2014). Broad and direct interaction between TLR and Siglec families of pattern recognition receptors and its regulation by Neu1. Elife.

[67] Sage AT, Walter LA, Shi Y, Khan MI, Kaneto H, Capretta A (2010). Hexosamine biosynthesis pathway flux promotes endoplasmic reticulum stress, lipid accumulation, and inflammatory gene expression in hepatic cells. Am J Physiol Endocrinol Metab.

[68] Zhou Y, Zhou H, Hua L, Hou C, Jia Q, Chen J (2021). Verification of ferroptosis and pyroptosis and identification of PTGS2 as the hub gene in human coronary artery atherosclerosis. Free Radic Biol Med.

[69] Linkermann A, Skouta R, Himmerkus N, Mulay SR, Dewitz C, De Zen F (2014). Synchronized renal tubular cell death involves ferroptosis. Proc Natl Acad Sci U S A.

[70] Tuo QZ, Lei P, Jackman KA, Li XL, Xiong H, Li XL (2017). Tau-mediated iron export prevents ferroptotic damage after ischemic stroke. Mol Psychiatry.

[71] Gao M, Monian P, Quadri N, Ramasamy R, Jiang X (2015). Glutaminolysis and Transferrin Regulate Ferroptosis. Mol Cell.

[72] Gao M, Yi J, Zhu J, Minikes AM, Monian P, Thompson CB (2019). Role of Mitochondria in Ferroptosis. Mol Cell.

[73] Chen G-H, Song C-C, Pantopoulos K, Wei X-L, Zheng H, Luo Z (2022). Mitochondrial oxidative stress mediated Fe-induced ferroptosis via the NRF2-ARE pathway. Free Radic Biol Med.

[74] Li N, Wang W, Zhou H, Wu Q, Duan M, Liu C (2020). Ferritinophagy-mediated ferroptosis is involved in sepsis-induced cardiac injury. Free Radic Biol Med.

[75] Seibt TM, Proneth B, Conrad M (2019). Role of GPX4 in ferroptosis and its pharmacological implication. Free Radic Biol Med.

[76] Ribas V, García-Ruiz C, Fernández-Checa JC (2014). Glutathione and mitochondria. Front Pharmacol.

[77] Koppula P, Zhang Y, Zhuang L, Gan B (2018). Amino acid transporter SLC7A11/xCT at the crossroads of regulating redox homeostasis and nutrient dependency of cancer. Cancer Commun (Lond).

[78] Dixon SJ, Patel DN, Welsch M, Skouta R, Lee ED, Hayano M (2014). Pharmacological inhibition of cystine-glutamate exchange induces endoplasmic reticulum stress and ferroptosis. Elife.

[79] Sato M, Kusumi R, Hamashima S, Kobayashi S, Sasaki S, Komiyama Y (2018). The ferroptosis inducer erastin irreversibly inhibits system x- and synergizes with cisplatin to increase cisplatin's cytotoxicity in cancer cells. Sci Rep.

[80] Ma L, Zhang X, Yu K, Xu X, Chen T, Shi Y (2021). Targeting SLC3A2 subunit of system XC- is essential for m6A reader YTHDC2 to be an endogenous ferroptosis inducer in lung adenocarcinoma. Free Radic Biol Med.

[81] He J, Liu D, Liu M, Tang R, Zhang D (2022). Characterizing the role of SLC3A2 in the molecular landscape and immune microenvironment across human tumors. Front Mol Biosci.

[82] Liu H, Deng Z, Yu B, Liu H, Yang Z, Zeng A (2022). Identification of SLC3A2 as a Potential Therapeutic Target of Osteoarthritis Involved in Ferroptosis by Integrating Bioinformatics, Clinical Factors and Experiments. Cells.

[83] Eyster CA, Cole NB, Petersen S, Viswanathan K, Früh K, Donaldson JG (2011). MARCH ubiquitin ligases alter the itinerary of clathrin-independent cargo from recycling to degradation. Mol Biol Cell.

[84] Digomann D, Linge A, Dubrovska A (2019). SLC3A2/CD98hc, autophagy and tumor radioresistance: a link confirmed. Autophagy.

[85] Lin X, Li S, Zhao Y, Ma X, Zhang K, He X (2013). Interaction domains of p62: a bridge between p62 and selective autophagy. DNA Cell Biol.

[86] Wang X, Terpstra EJM (2013). Ubiquitin receptors and protein quality control. J Mol Cell Cardiol.

[87] Pattarabanjird T, Marshall M, Upadhye A, Srikakulapu P, Garmey JC, Haider A (2022). B-1b Cells Possess Unique bHLH-Driven P62-Dependent Self-Renewal and Atheroprotection. Circ Res.

[88] Zhao J, Hu B, Xiao H, Yang Q, Cao Q, Li X (2021). Fucoidan reduces lipid accumulation by promoting foam cell autophagy via TFEB. Carbohydr Polym.

[89] Cheng Y, Pan X, Wang J, Li X, Yang S, Yin R (2020). Fucoidan Inhibits NLRP3 Inflammasome Activation by Enhancing p62/SQSTM1-Dependent Selective Autophagy to Alleviate Atherosclerosis. Oxid Med Cell Longev.

[90] Kim PK, Hailey DW, Mullen RT, Lippincott-Schwartz J (2008). Ubiquitin signals autophagic degradation of cytosolic proteins and peroxisomes. Proc Natl Acad Sci U S A.

