# Supplementary material for: Metabolite Neu5Ac triggers SLC3A2 degradation promoting vascular endothelial ferroptosis and aggravates atherosclerosis progression in ApoE-/-mice
Source: Theranostics. 2023 Sep 4;13(14):4993–5016. doi: 10.7150/thno.87968 (PMC10526676; doi:10.7150/thno.87968)
Supplement: Supplementary file 1 — Supplementary figures. [file thnov13p4993s1.pdf]

Supplementary materials

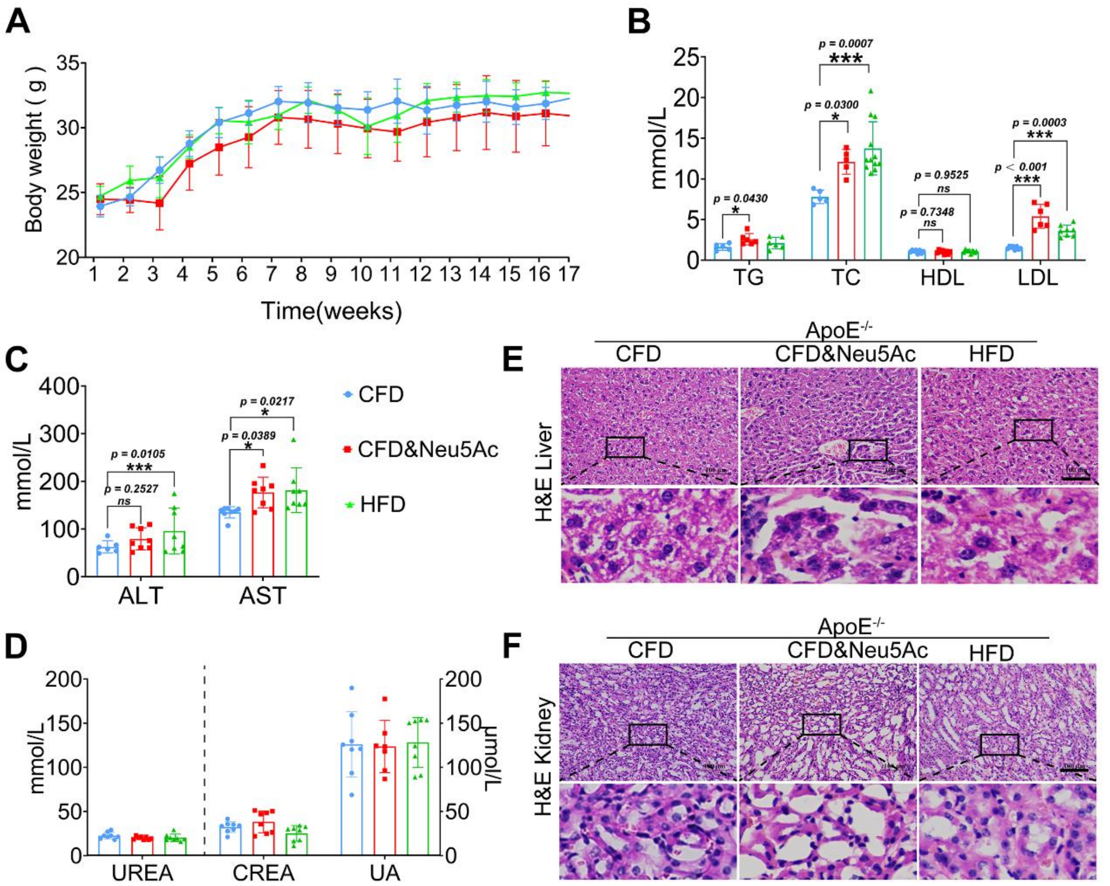

Figure S1 Intraperitoneal injection with Neu5Ac induces liver and kidney injury in ApoE$^{-/-}$ mice. (A) Bodyweight showed no significant changes for 17 weeks in each group. (B) TC, TG, HDL-C, and LDL-C levels measured by biochemical analyzer, showing increased TC, TG, LDL levels in HFD-fed and Neu5Ac-injected groups compared with mice in CFD-fed group. (C) Liver function indicators (AST, ALT) were measured by biochemical analyzer, showing higher in HFD-fed and Neu5Ac-injected groups than CFD-fed group. (D) Kidney function indicators (UREA, CREA, UA) were measured by biochemical analyzer. (E, F) Liver and kidney H&E staining, exhibiting obvious tissue damage in HFD-fed and Neu5Ac- injected groups. Statistical analysis was performed by one-way ANOVA followed by Tukey test for A-D. TC: Total cholesterol, TG: Triglycerides, HDL-C: High-density lipoprotein cholesterol, LDL-C: Low-density lipoprotein cholesterol.

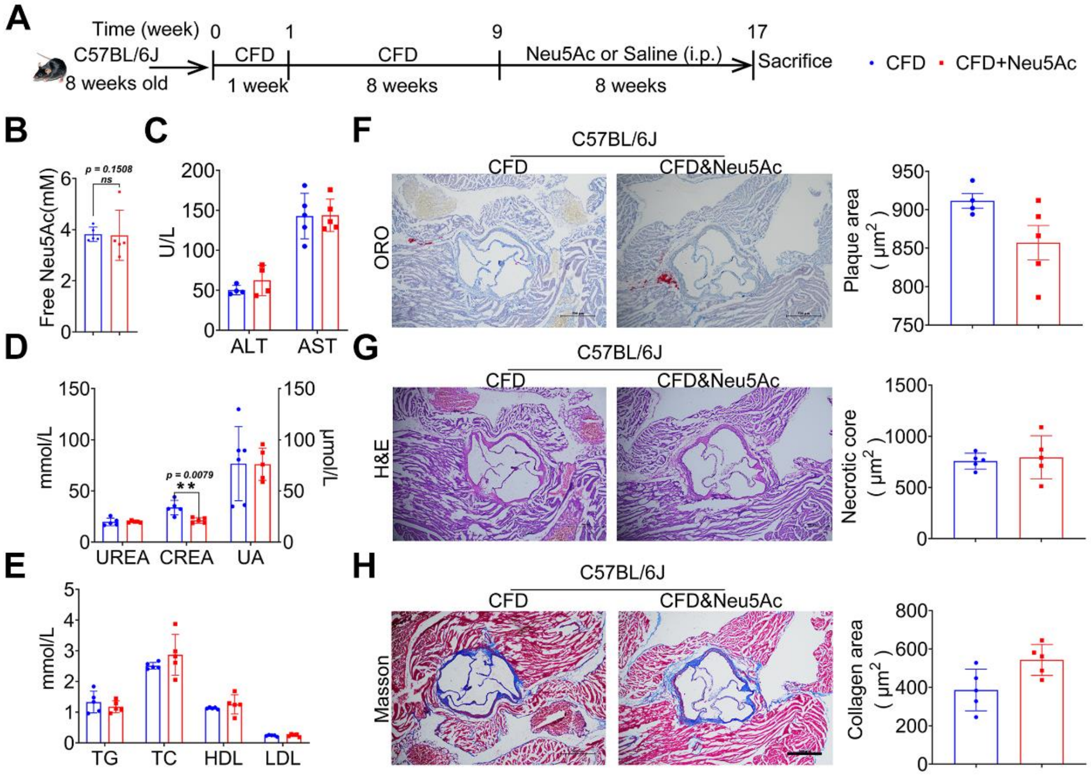

Figure S2 Normal metabolic conditions C57BLJ/6 mice are less sensitive to Neu5Ac compared to ApoE$^{-/-}$ mice.

(A) Experimental design of *C57BL/6J* mice. *C57BL/6J* mice fed a CFD or HFD for 8 weeks, then intraperitoneally injected with Neu5Ac (60 mg/kg/d) or saline (60 mg/kg/d) for 8 weeks. (B) Free Neu5Ac in the plasma of *C57BL/6J* mice had no significant changes (n = 5 per group). (C) Liver function indicators (AST, ALT) were measured by biochemical analyzer, showing no significant changes. (D) kidney function indicators (UREA, CREA, UA) were measured by biochemical analyzer. (E) TG, TC, LDL, and HDL were detected by biochemical analyzer. (F) Representative Oil Red O-stained aortic sinus, with quantitative data in the right panel. Scale bars = 500 μm (n > 3 per group). (G) H&E staining of lesions of the aortic sinus, with quantitative data in the right panel. Scale bars = 500 μm (n > 3 per group). (H) Representative images of the aortic sinus stained with Masson's trichrome stain, with quantitative data in the right panel. Scale bars = 500 μm (n > 3 per group). Statistical analysis was performed by Student t test for B-H.

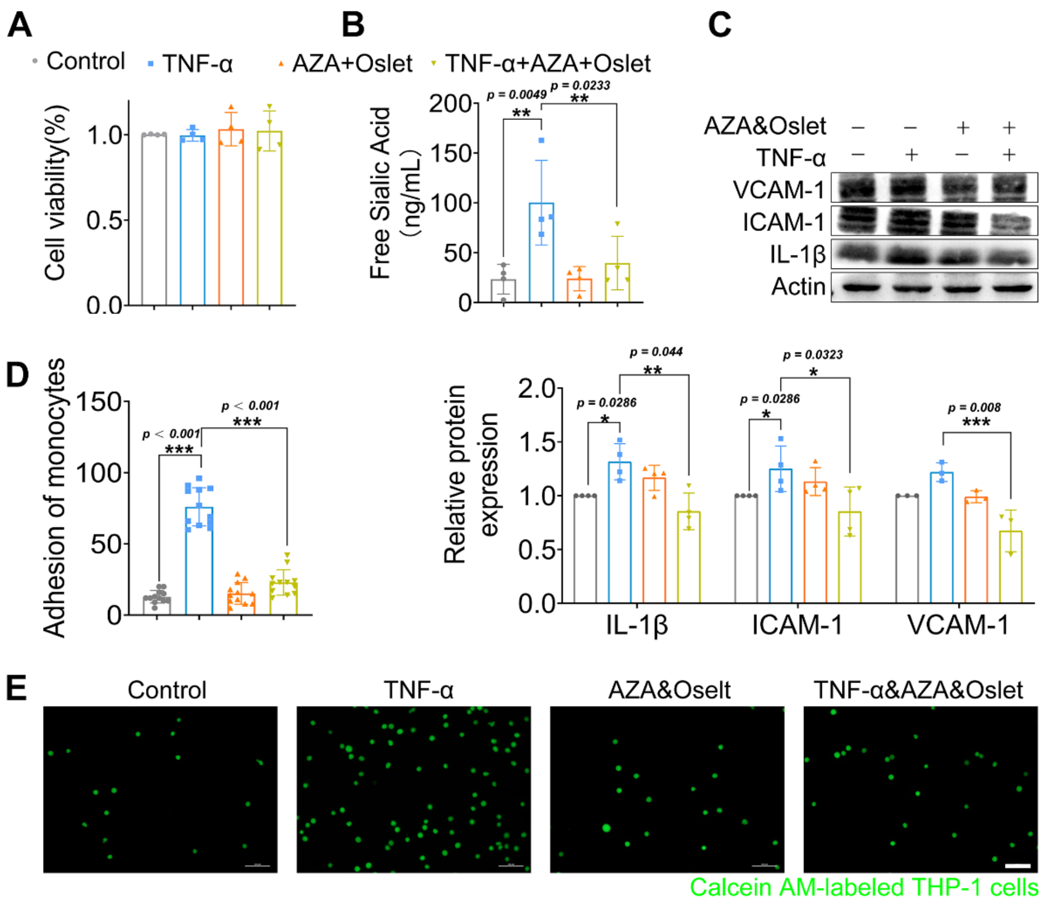

Figure S3 Elevated levels of endogenous Neu5Ac also induces inflammatory injury in vascular endothelial cells.

HUVECs treated with TNF-α (200 ng/mL), Azaserine (25 μM) & Oseltamivir (200 μM), or TNF-α & Azaserine & Oseltamivir for 12 h. (A) HUVECs viability was measured by CCK-8 assay, showing no significant changes (n = 4 per group). (B) LC-MS detection of free Neu5Ac in HUVECs medium, which were increased after TNF-α treated and decreased following co-treated with AZA & Oslet (n = 4 per group). (C) Western blotting showed TNF-α induced increased IL-1β, ICAM-1 protein levels, which were decreased by AZA & Oslet co-treated. The quantification data was shown at the bottom (n = 4 per group). (E) Representative images of monocyte-endothelial adhesion, with quantification data at the top (D) (n ≥ 3 per group). Statistical analysis was performed by one-way ANOVA followed by the Tukey test for A-D.

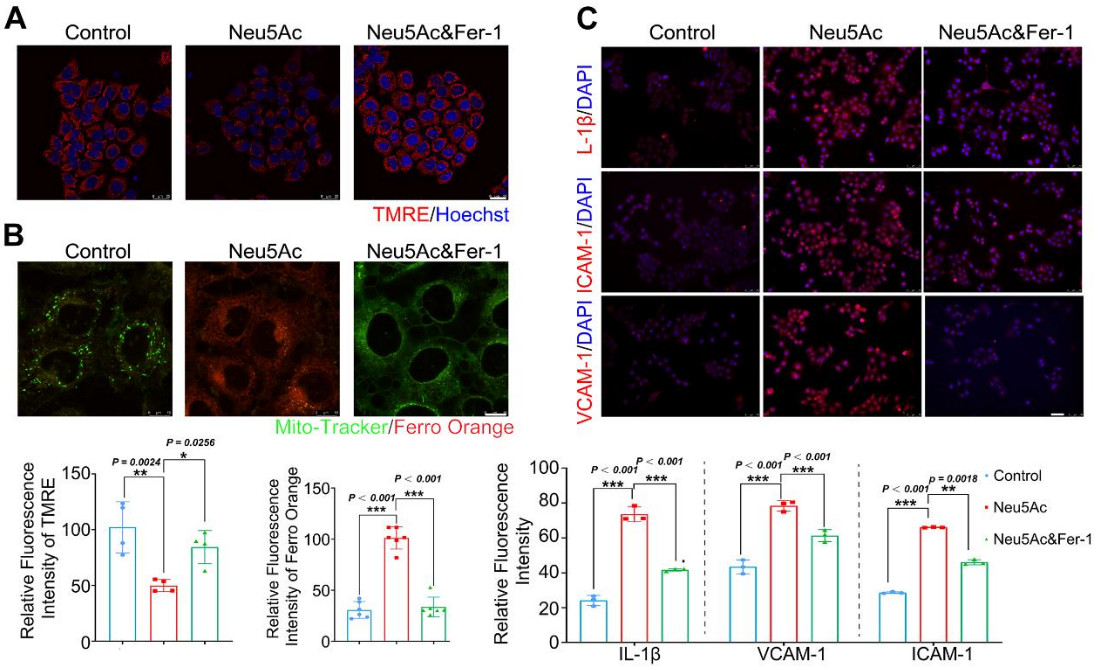

Figure S4 Inhibition of ferroptosis restores mitochondrial function and suppresses vascular endothelial inflammation.

HUVECs were treated with Neu5Ac (20 mM) or Neu5Ac (20 mM) & Fer-1(1 μM) for 24 h. (A) The representative images of immunofluorescence staining of TMRE (red), and Hoechst (blue) are shown, with the quantification data at the bottom. Scale bar = 25 μm (n = 4 per group). (B) Colocalization of Mito-Tracker (green) and Ferro Orange (red) in HUVECs, with the quantification data at the bottom. Scale bar = 10 μm (n = 6 per group). (C) Immunofluorescence staining of IL-1β, ICAM-1and VCAM-1, with the quantification data at the bottom. Scale bar = 50 μm (n = 3 per group). Statistical analysis was performed by one-way ANOVA followed by the Tukey test for A-C.

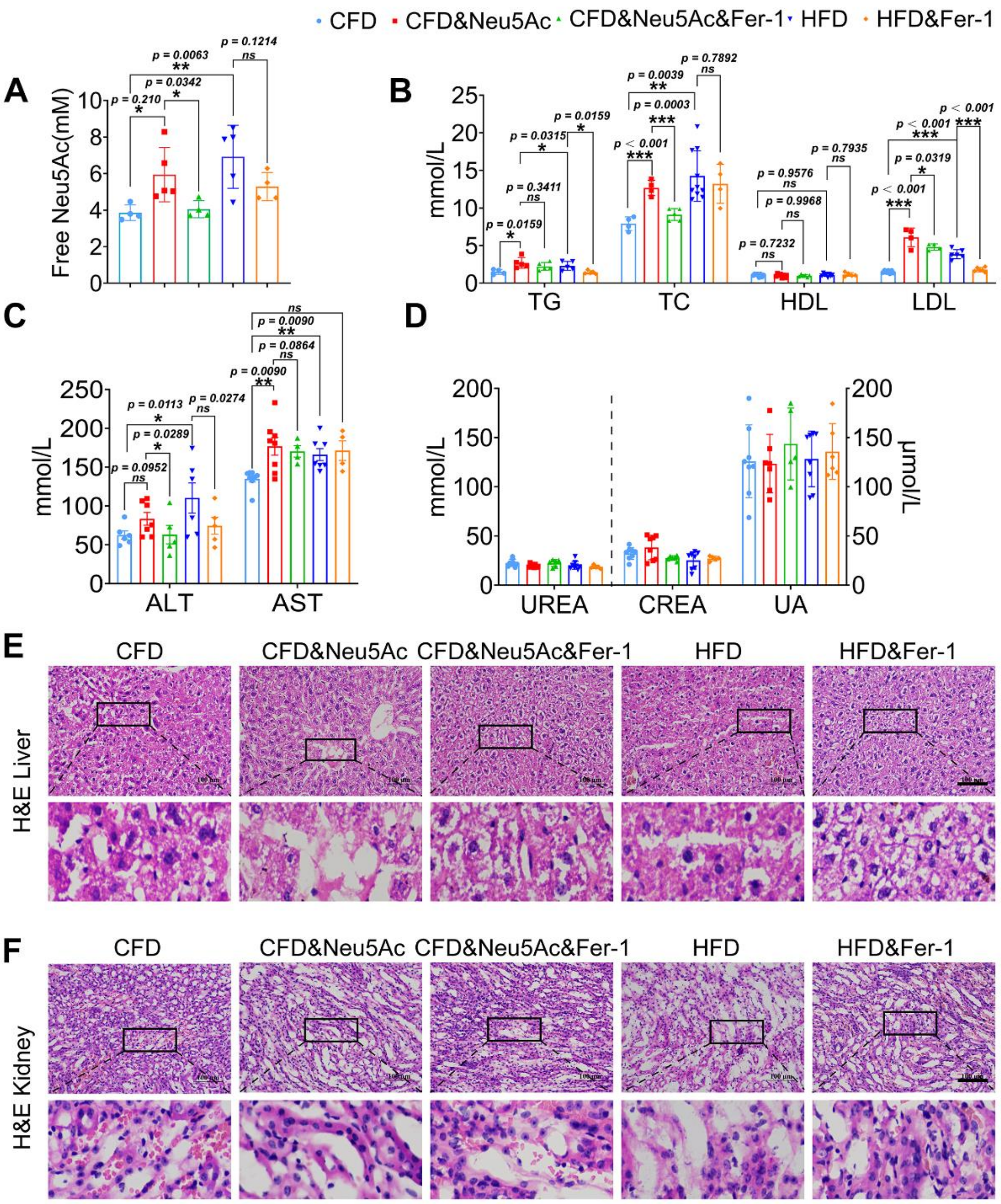

Figure S5 Effect of Fer-1 on lipids levels as well as liver and kidney function in ApoE$^{-/-}$ mice injecting Neu5Ac or HFD feeding.

(A) Free Neu5Ac in the plasma were increased in HFD-fed and Neu5c-injected (60 mg/kg/d) groups, which were decreased by co-injecting with Fer-1 (10 mg/kg/d) ($n \geq 4$ per group). (B) TG, TC, LDL, and HDL detected by biochemical analyzer, demonstrating elevated TC, TG, LDL levels in HFD-fed and Neu5Ac-injected groups, which were inhibited by co-injected with Fer-1 (10 mg/kg/d) ($n > 3$ per group). (C, D) Liver function indicators (AST, ALT) and kidney function indicators (UREA, CREA, UA) were measured by biochemical analyzer, showing no significant changes following co-injecting with Fer-1 ($n > 3$ per group) (E, F) Liver and kidney H&E staining. Apart from the CFD feeding group, other groups showed liver, and kidney injury ($n > 3$ per group). Statistical analysis was performed by one-way ANOVA

followed by the Tukey test for A-D.

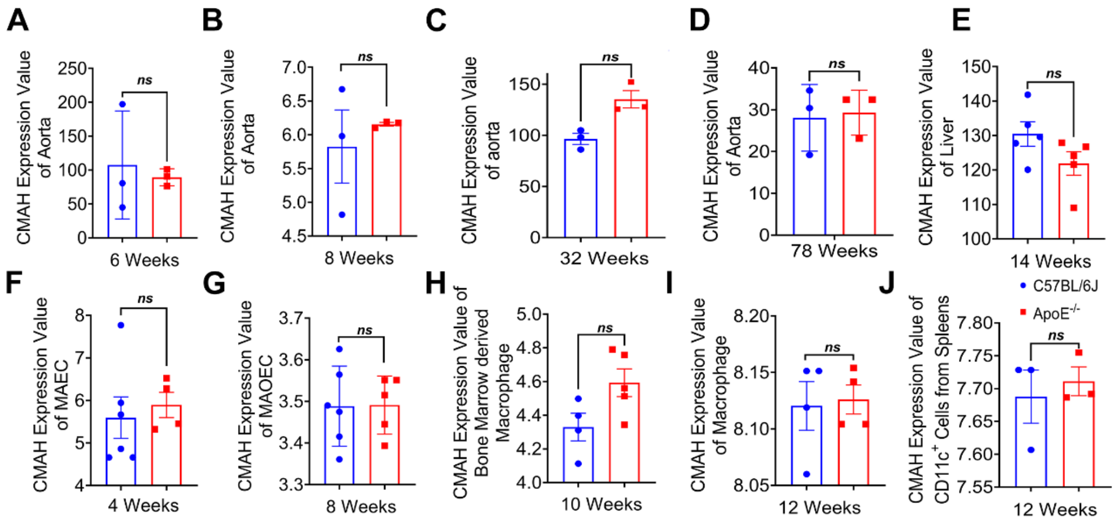

Figure S6 In AS process, with no obvious difference in the expression level of CMAH between ApoE$^{-/-}$ mice and C57BL/6J mice.

CMAH expression value of aorta in *C57BL/6J* and *ApoE$^{-/-}$* mice (A) at 6 weeks in GSE10000, (B) CMAH at 8 weeks in GSE83112, (C) at 32 weeks in GSE2372, (D) at 78 weeks in GSE10000. CMAH expression value of liver at 14 weeks in GSE28125. (F) CMAH expression values in mouse aortic endothelial cells (MAEC) at 4 weeks in GSE39264. (G) CMAH expression values in Primary mouse aorta endothelial cells (MAOEC) at 8 weeks in GSE39264. (H) CMAH expression values in bone marrow derived macrophage at 10 weeks in GSE143533. (I) CMAH expression values in macrophage at 12 weeks in GSE70126. (J) CMAH expression values in CD11c^{+} cells from spleens at 12 weeks in GSE191044.

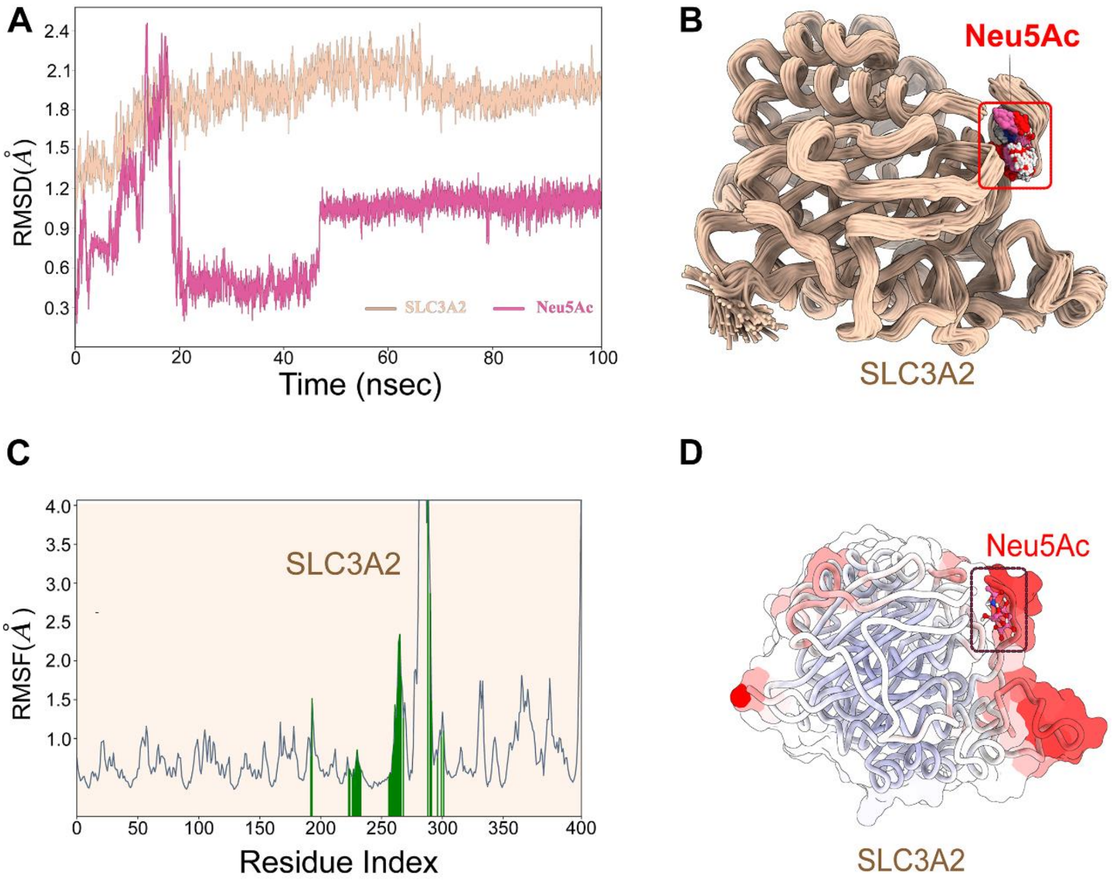

Figure S7 Molecular dynamics simulation of SLC3A2-Neu5Ac complex.

(A-B) RMSD analysis of SLC3A2, and Neu5Ac during 100-ns MD simulations. (C-D) RMSF analysis of SLC3A2, and Neu5Ac. RMSD: Root Mean Square Deviation, RMSF: Root Mean Square Fluctuation.

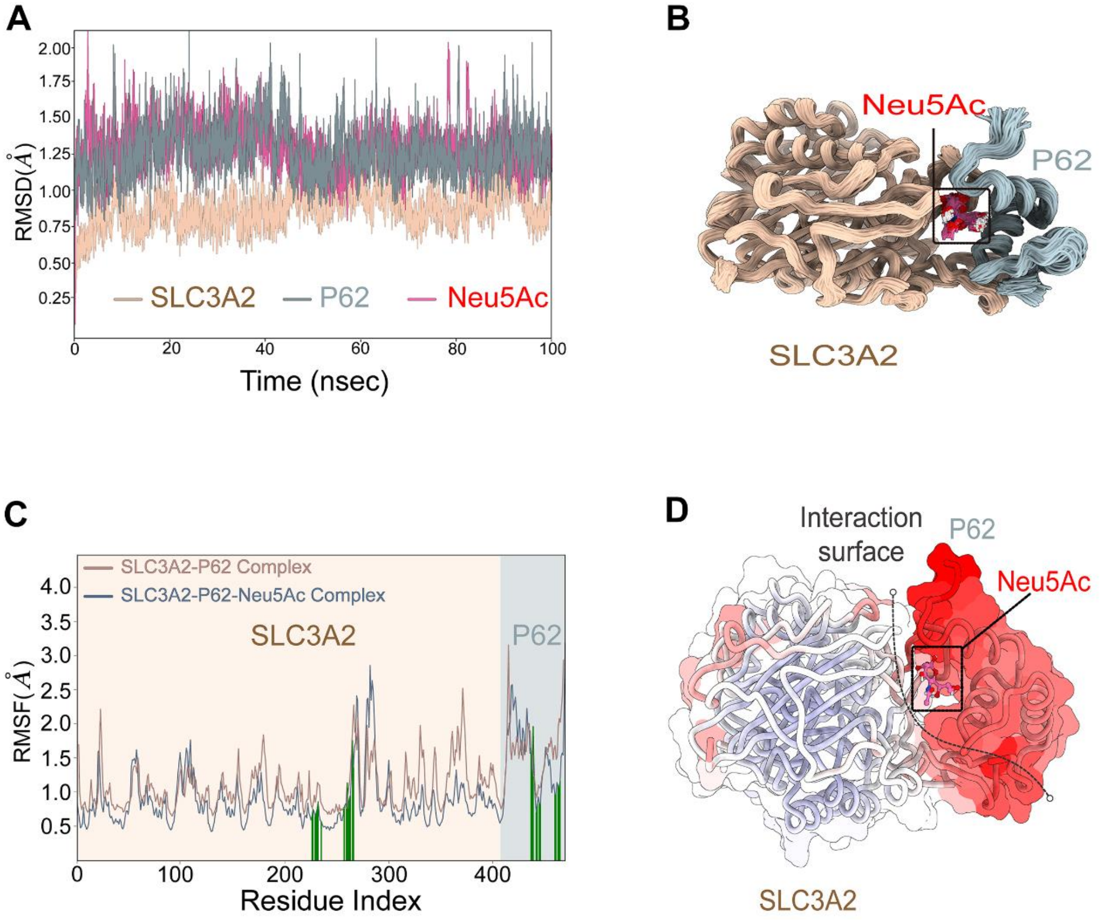

Figure S8 Molecular dynamics simulation of SLC3A2-Neu5Ac-P62 complex.

(A-B) RMSD analysis of SLC3A2, Neu5Ac, and P62 during 100-ns MD simulations. (C-D) RMSF analysis of SLC3A2, Neu5Ac, and P62. RMSD: Root Mean Square Deviation, RMSF: Root Mean Square Fluctuation
